# Supplementary material for: Sex and friendship in a multilevel society: behavioural patterns and associations between female and male Guinea baboons
Source: Behav Ecol Sociobiol. 2016 Jan 22;70:323–36. doi: 10.1007/s00265-015-2050-6 (PMC4748025; doi:10.1007/s00265-015-2050-6)
Supplement: Supplementary file 1 — (DOCX 731 kb) [file 265_2015_2050_MOESM1_ESM.docx › 265_2015_2050_AuthorFeedback_1.pdf]

DOI: 10.1007/s00265-015-2050-6

File Correction Details

Correction is made. No of Corrections: 76

Online Correction Link : **http://eproofing.springer.com/journals/index.php?token=05uhjh_f0Qplq--CoFd9gzxMHNtF5U3C**

Image Annotation Details

No Details Found

Attached File Details

Fig._1ab_5m_2m_networks.png Fig._2a_Pgroom.png Fig._2b_Pgreet.png Fig._2c_Paggress.png Fig._2d_Pinfhandle.png Fig._3_hinde_ppt.png Fig._4_Pgreetir 5_temporal_dynamics.png Fig._6_tenure_mod.png 265_2015_2050_MOESM1_ESM_revised.docx

Query Details

1. **Please check Article Note if presented and captured correctly.**

   I have checked the article note and made changes directly to the text.

2. **“Kopp et al. 2014” is cited in text but not given in the reference list. Please provide details in the list or delete the citation from the text.**

   The correct citation:

Kopp GH, Ferreira da Silva MJ, Fischer J, Brito JC, Regnaut S, Roos C, Zinner D (2014) The Influence of Social Systems on Patterns of Mitochondrial DNA Variation in Baboons. Int J Primatol 35:210–225

3. **“Soltis et al. 2001” is cited in text but not given in the reference list. Please provide details in the list or delete the citation from the text.**

   The correct citation:

Soltis J, Thomsen R, Takenaka O (2001) The interaction of male and female reproductive strategies 760 and paternity in wild Japanese macaques, *Macaca fuscata*. Anim Behav 62:485–494

4. **Table 2: Please check if data are presented correctly.**

   The data are presented correctly.

5. **Table 3: FYI: Indicators in the table were changed to superscripted letters as per journal instruction.**

   Thank you.

6. **“Seyfarth and Cheney 2012” is cited in text but not given in the reference list. Please provide details in the list or delete the citation from the text.**

   The correct citation:

Seyfarth RM, Cheney DL (2012) The Evolutionary Origins of Friendship. Annu Rev Psychol 63:153–177

7. **“Schreier and Swedell 2012” is cited in text but not given in the reference list. Please provide details in the list or delete the citation from the text.**

   The correct citation:

Schreier AL, Swedell L (2012) Ecology and sociality in a multilevel society: Ecological determinants of spatial cohesion in hamadryas baboons. Am J Phys Anthropol 148:580–588

8. **“Müller 1980” is cited in text but not given in the reference list. Please provide details in the list or delete the citation from the text.**

   The correct citation:

Muller MN, Kahlenberg SM, Thompson EM, Wrangham RW (2007) Male coercion and the costs of promiscuous mating for female chimpanzees. Proc R Soc B Biol Sci 274:1009–1014

9. **Reference [Schreier AL, Swedell L (2009) The fourth level of social structure in a multi-level society: ecological and social functions of clans in hamadryas baboons. Am J Primatol 71:948–955 2012] was provided in the reference list; however, this was not mentioned or cited in the manuscript. As a rule, all the references given in the list of references should be cited in the body of a text. Please provide the location of where to insert the reference citation in the main body text.**

   This was an error the correct citation is below:

Schreier AL, Swedell L (2012) Ecology and sociality in a multilevel society: ecological determinants of spatial cohesion in hamadryas baboons. Am J Phys Anthropol 148:580–588

10.1007/s00265-015-2050-6

Sex and friendship in a multilevel society: behavioural patterns and associations between female and male Guinea baboons

Adeelia S. Goffe [1,*]

Phone +49 (0)551 3851378 Email agoffe@dpz.eu

Dietmar Zinner [1]

Julia Fischer [1]

[1] Cognitive Ethology Laboratory, German Primate Center, Kellnerweg 4, 37077 Göttingen, Germany

Abstract

One key question in social evolution is the identification of factors that promote the formation and maintenance of stable bonds between females and males beyond the mating context. Baboons lend themselves to examine this question, as they vary in social organisation and male-female association patterns. We report the results from the first systematic observations of individually identified wild female Guinea baboons. Guinea baboons live in a multilevel society with female-biased dispersal. Although several males could be found within 5 m of females, each female chiefly associated with one "primary" male at the 2 m distance. Social interactions occurred predominantly with the primary male, and female reproductive state had little influence on interaction patterns. The number of females per primary male varied from 1 to 4. During the 17-month study period, half of the females transferred between different males one or multiple times. A subset of females maintained weaker affiliative nonsexual relationships with other "secondary" males. Units composed of primary males with females, and occasional secondary males, apparently form the core of the Guinea baboon society. The social organisation and mating patterns of Guinea and hamadryas baboons may have a common evolutionary origin, despite notable differences in relationship quality. Specifically, Guinea baboon females appear to have greater leverage in their association patterns than hamadryas baboon females. Although we cannot yet explain the lack of overt male control over females, results generally support the notion that phylogenetic descent may play an important role in shaping social systems.

Keywords

Papio papio
Intersexual relationships
Mating system
Pair bond
Social organisation

Communicated by R. I. M. Dunbar

Significance statement

The wide range of variability in intersexual relationships across species makes the identification of key factors driving relationship formation and maintenance challenging. From the female perspective, we provide comprehensive data on the intersexual relationships and spatial associations of wild Guinea baboons, a little-studied baboon species with a multilevel social system, female-biased dispersal and high levels of male-male tolerance. Our data indicate that one or more females are closely bonded to a primary male with whom they affiliate and copulate. The occurrence and intensity of these relationships varied minimally with the oestrous phase of females. Females exhibited spatial freedom, indicating some element of female choice. A subset of females maintained weaker nonsexual friendships with secondary males. ~~Although superficially similar, there may be a common evolutionary origin for the social organisation and mating system of hamadryas and Guinea baboons.~~Superficial similarities in the social organisation and mating systems of hamadryas and guinea baboons hint at a common evolutionary origin.

AQ1

Electronic supplementary material

The online version of this article (doi: 10.1007/s00265-015-2050-6~~-~~) contains supplementary material, which is available to authorized users.

Introduction

Social relationships between females and males vary widely in their temporality, intensity and modes of expression. According to socio-ecological theory, males compete for access to fertile females, while females may aim for high-quality males, access to resources and/or paternal care (Emlen and Oring 1977; Greenwood 1980). ~~The m~~Males' ability to monopolise females depend on a number of factors, including the size and distribution of females' home ranges, the distribution of feeding patches and food quality, or the length and synchrony of the females' breeding cycle (Davies and Lundberg 1984; Ridley 1986; Sterck et al. 1997). Thus, for the majority of mammals, social interactions between females and males are restricted to courtship and mating. This is the most pronounced in solitary foraging species in which females and males come together for brief periods to mate (e.g. honey badger, *Mellivora capensis*: Begg et al. 2006; orang-utan, *Pongo pygmaeus*: Mitani 1990; polar bear, *Ursus maritimus*: Molnar et al. 2008). In gregarious species that live in bisexual groups, there is the potential for social contact at all phases of the female reproductive cycle, but intersexual interactions may still vary with changes in female reproductive state (e.g. eastern chimpanzee, *Pan troglodytes schweinfurthii*: Muller et al. 2007; spotted hyaena, *Crocuta crocuta*: Szykman et al. 2003, 2007; Grevy's zebra, *Equus grevyi*: Sundaresan et al. 2007).

Baboons (genus *Papio*) lend themselves for investigating the link between mating and relationship patterns in societies with different social organisations. Commonly known as "savannah" baboons, chacma (*P. ursinus*), olive (*P. anubis*) and yellow baboons (*P. cynocephalus*) live in multi-male multi-female groups with female philopatry and male dispersal. Their mating system can be regarded as polygynandrous, whereby male rank predicts mating success and reproductive skew (Swedell 2011). Intersexual associations are conspicuous and vary with female reproductive state. During consortships, oestrous females and males stay in close proximity (Swedell 2011). Lactating females with dependent infants maintain affiliative "friendships" with specific males, most likely as a measure against harassment and infanticide risk (Lemasson et al. 2008; Palombit 2009).

In contrast, hamadryas baboons (*P. hamadryas*) have a multilevel social system in which all females, regardless of reproductive state, maintain close proximity to specific males (the leader males), resulting in the formation of one-male units (OMUs). One or more females spatially, socially and sexually affiliate with one male, resulting in the formation of OMUs. OMUs are spatially segregated from other OMUs, partly through male enforcement (Kummer 1968; Swedell and Schreier 2009) and form the social core of these societies (Kummer 1968). Some OMUs may also have follower males, which are significantly less social with females than are leader males (Kummer 1968; Swedell 2006; Pines et al. 2011; Chowdhury et al. 2015). Multiple OMUs together form higher nested social levels, which vary in size and composition (clan, band, troop; see Swedell 2011 for review). Furthermore, in contrast to savannah baboons, hamadryas baboon males are predominantly philopatric, but both sexes may disperse (Swedell et al. 2011; Städele et al. 2015).

Until recently, much less was known about Guinea baboons (*P. papio*). Observations of male-male association patterns revealed that they live in a multilevel social system in which several males form parties, which in turn regularly aggregate into gangs (Patzelt et al. 2014). Males exhibit high levels of tolerance and maintain relationships with other males (Patzelt et al. 2014). Genetic evidence suggests that, similar to hamadryas baboons, there is female-biased dispersal (Kopp et al. 2014, 2015). To date, work regarding intersexual relationships had been conducted on either unhabituated populations in the wild or on captive groups and had led to conflicting conclusions regarding the social system of this species. Some researchers proposed a multi-male multi-female social system similar to that of ~~some~~several savannah baboon populations (Sharman 1982). Others assumed weak substructuring in which OMUs are sometimes present (Dunbar and Nathan 1972) or a multilevel social system containing OMUs (Boese 1973; Maestripieri et al. 2005, 2007; Galat-Luong et al. 2006).

AQ2

Here, we present data from the first systematic observations from individually identified female Guinea baboons, with respect to their association and interaction patterns with males. The goal of this study is to clarify the intersexual social relationships and mating patterns of this species. Of primary interest was whether female-male association patterns conformed generally to the savannah baboon model, where intersexual relationships are mainly confined to the oestrous period and lactation, or whether females associated with males throughout their reproductive cycle, as in the case of hamadryas baboons. We conducted social network analyses based on proximity scans to identify substructures within the social group and investigated interaction patterns between females and males, with a specific interest in the temporal dynamics of intersexual associations.

Methods

Field site and study subjects

Research took place at the Centre de Recherche de Primatologie (CRP) field station in the Parc National du Niokolo Koba, Senegal (as described in Maciej 2013) from January 2012 to July 2013. The Guinea baboon population around the CRP field station consisted of >400 individuals, comprising 5–7 gangs varying in degree of habituation. We observed members of the Mare gang, which included three parties (party IDs 4, 9 and 10), because they were the best habituated gang at that time. At the onset of the study, all individuals in the focal gang could be followed by the observer (ASG) from a distance of 10–12 m. Other gangs in the community were not as well habituated, but could be followed easily at a distance of ≥20 m. By the onset of focal sampling in April 2012, all individuals in the Mare gang could be followed even through dense vegetation at a distance of <5 m, if necessary, and by May 2012 it was possible to observe this gang when feeding/travelling in aggregations of >200 baboons without causing obvious disruption. Gang size and composition varied during the study period. The study gang consisted of 15–16 adult females, 0–2 subadult females, 11–12 adult males and 3–6 subadult males. Variation in gang composition was due to maturation, mortality and migration events.

Data collection

Our study involved focal observations of wild animals in the field making it impossible to use blinded methods to record the data. Electronic forms for data collection were created using Pendragon 5.1.2 software (Pendragon Software Corporation, USA) and run on HP Tungsten Palm E2 handhelds (Hewlett-Packard Company, USA). As a part of the daily census, a single observer, ASG, recorded the presence and health status for all individuals in the study group, with female reproductive status noted for all focal females (Gauthier 1999; Higham et al. 2009). Females observed to suckle-dependent offspring we categorised as lactating; pregnant females were distinguished by reddening of the anogenital area (AGA) and the paracallosal skin (PCS). Cycling females were partitioned into four categories: C0 (an absence of swelling in the AGA and PCS), C1 (a small vertical swelling of the AGA), C2 (a medium (vertical and horizontal) swelling of the AGA and a small swelling of the PCS) and C3 (a full outward distention of both the AGA and the PCS; however, the width at peak swelling did not extend beyond the outer extremities of the ischial callosities as it

does in other *Papio* species (Gauthier 1999; Higham et al. 2009)).

Ad libitum data on intersexual grooming, greeting, copulation and aggressive interactions were collected during ~2100 h over the course of 489 observation days (2012 = 328 and 2013 = 161) from 06:00 to 13:00 and 15:00 to 19:00. Focal data (totalling 1262 completed samples of 30 min each) were collected over the course of 256 study days from 16 adult females from April to August 2012 and December 2012 to June 2013.

As proximity distances have been suggested as good indicators of social relationships (Kummer 1968; Fernando and Lande 2000; Lusseau 2003), four scans were conducted per 30 min follow in order to record the location of all adult and subadult males within 1–2 m (henceforth referred to as 2 m) and ≥2–5 m (henceforth referred to as 5 m) of the focal female. One scan was conducted at the start of each focal protocol, with subsequent scans occurring at 10 min intervals~~;~~. ~~a~~A total of 5048 proximity scans were analysed to assess spatial proximity, irrespective of the occurrence or quality of social interactions. From previous studies, we knew that spatial and interaction networks do not necessarily correlate (Castles et al. 2014; Patzelt et al. 2014); although social interaction is contingent upon spatial proximity, the reverse is not necessarily true.

Focal observations of 30 min in duration were conducted for each female 1–3 times per week during morning and afternoon sessions, throughout which the occurrence of all approaches (within 2 m), retreats, supplants (approach-retreat interactions in which individuals maintain close proximity for less than 5 s), grooming, greeting, aggression and copulation events were recorded (Altmann 1974). Grooming bout durations were recorded to the closest second and involved either bilateral or unilateral grooming of one or both partners. Bouts were defined as episodes that were not interrupted for more than 2 min or by an active social interaction with another individual. Greetings, approach-retreat interactions often involving affiliative "grunt" vocalisations (Maciej et al. 2012), were also recorded and involved at least one element of contact (e.g. ventral embrace, genital touching or sniffing, or mounting). As aggression events varied in duration and were often polyadic in nature, aggression events were determined to have ended when one of the participants retreated from the other or affiliative behaviours were observed between the two individuals. Copulations were recorded for all tumescent (with a sexual swelling) adult females ad libitum; in order to distinguish between socio-sexual and reproductive sexual behaviours, only full mountings that occurred (most likely) with intromission while a female was tumescent were recorded as copulations. Mounts with non-tumescent females were categorised as greetings.

Data analyses

All statistical analyses were conducted in the R environment version 3.1.2 (R Core Team 2014) and RStudio interface (R Studio 2012). The individual citations for functions and packages utilised are given below.

Intersexual network structure

As grouping patterns varied throughout the study period, we confined the social network analysis to a stable 2-month period from April to June 2012. The analysis is based on 1360 scan samples, ranging from 84 to 96 per female, for two different proximity distances: 5 and 2 m. We calculated degree centrality, density and applied community identification algorithms (spin glass and walktrap). Degree centrality was used to determine the number of immediate neighbours for each individual and we then ran a Mann-Whitney *U* test with the function *wilcox.test* in the stats package (R Core Team 2014), to determine whether males differed in terms of the number of their female partners. Proximity networks were undirected and weighted in order to visualise the varying intensity of connections. The success of intersexual pairings was assessed for each female individually by comparing the subgroup assignment to the male node, which had the highest number of connections. Figures were generated using the Fruchterman Reingold layout (Fruchterman and Reingold 1991), and the calculation of network metrics was performed in R using available functions in the package igraph (Csárdi and Nepusz 2006): *graph.strength*, *graph.density*, *spinglass.community* and *walktrap.community*. Additional details regarding these methods are included in the supplementary material.

Identification of male partners

In order to assess if females have preferred male associates, we analysed 5- and 2-m proximity scans collected over the entirety of the study period (ranging from 160 to 344 scan samples for each female). We individually assessed whether each female revealed preferential associations with specific males, which included 20 subadult and adult males, using a Friedman average rank test, a nonparametric test for repeated measures (Friedman 1940; Demšar 2006). We then used the Nemenyi post hoc test to test the difference in rank for all pairwise comparisons (Demšar 2006); see details in the supplementary material. Tests were conducted using the functions *friedman.test* from the stats package (R Core Team 2014) and *posthoc.friedman.nemenyi.test* in the PMCMR package (Pohlert 2014).

Two-metre scans collected throughout the course of the study were used to visualise weighted proximity networks using the package igraph (Csárdi and Nepusz 2006) with the Fruchterman Reingold layout, which clusters more strongly connected sets of nodes together (Fruchterman and Reingold 1991). As there may be temporal changes in intersexual associations, data were pooled every 2 weeks and the top male for each female (the male who was recorded most often within 2 m) was assigned as her "primary male"; other males were categorised as "secondary" if they were observed within 2 m or "unaffiliated" (with any female) if they were never observed within 2 m of a female. This method resembles the one used for determining preferred intersexual partners in Grevy's zebra (Sundaresan et al. 2007).

In order to determine if females were more likely to interact with males of different status categories (primary, secondary and unaffiliated), we looked at the occurrence of social contacts during focal observations. Social behaviours of interest included grooming, greeting, aggression and infant handling. Every focal observation (40 to 86 samples per female) received a yes/no score for each of the possible 20 subadult and adult males. We then ran generalized linear mixed models (GLMMs) (function *glmer* from the statistical package lme4; Bates et al. 2013) controlling for female and male identity, as well as the random slope for status and male identity. Due to the small amount of variability in the number of dyads observed to copulate, it was not possible to compare this behaviour statistically.

Directionality of relationship maintenance

From 1262 focal samples, we determined the overall percentage of approaches performed by females towards their primary and secondary males. In addition, we calculated the Hinde index (Hinde and Atkinson 1970; Hinde 1977) in order to determine which individual was responsible for maintaining proximity and, potentially, female social partner choice (Soltis et al. 2001). The index was calculated using the equation:

$$\text{Hinde index (HI)} = A_{\mathrm{f}} - R_{\mathrm{f}}$$

AQ3

where *A* is the proportion of approaches performed by the female and the *R* is the proportion of retreats performed by the female; supplants were not included in the calculations. Proportions were calculated from the total number of approaches or retreats a female experienced. HI scores range from −1, indicating male-driven relationships to +1, suggesting female-driven relationships. As only dyads having ≥10 approach-retreat interactions were included, one dyad containing a primary male and 16 dyads containing secondary males were excluded due to a low number of interactions. We tested the variability between dyads containing different male status categories while controlling for female and male identity with a GLMM using the function *lmer*.

Temporal dynamics of female-male associations

In order to assess the temporal stability of intersexual relationships during periods when focal scans were not collected, ad libitum grooming, greeting, copulation and aggression data were used. For females who interacted with more than one male on a regular basis, it was necessary to observe an interaction that was not surreptitious (that is, an interaction which occurred when the primary male was in the direct line of sight of the pair) in order for her to be recorded as changing from one primary male to another. Otherwise, it was assumed that the identity of the primary male had not changed.

Female reproductive state and intersexual relationships

To investigate the influence of female reproductive state on the probability of grooming, greeting or aggression occurring between females and their primary males, we ran three GLMMs (Baayen et al. 2008), with binomial error structure (occurrence yes/no). The predicted probability (based on the proportion of number of observations) of grooming, greeting and aggression occurring was modelled based on focal samples from 16 females observed in 1–6 reproductive states. In order to investigate the variability in the intensity of social interactions, a second set of models including only the observations during which grooming or greeting occurred (227 and 345 focal observations, respectively). The grooming duration and the greeting frequency per 30-min focal observation were assessed using GLMMs with Gaussian and Poisson error structures, respectively. In the Poisson model utilising counts of the number of greeting events, a log-transformed offset term was used (the number of focal observations per female).

Comparisons of the estimates of the models based on all data with estimates with effects excluded individually revealed that all the models were relatively stable. Variance inflation factors (Field 2009) for both variables in all three models did not indicate that collinearity was an issue; none of the data sets were found to be overdispersed. All models were implemented in R using the functions *lmer* and *glmer* in the package lme4 (Bates et al. 2013). Female and male identity were always included as random effects (Kreft and ~~D~~de Leeuw 1998).

Results

Intersexual network structure

~~Males~~Females were located within 5 m of ~~fe~~males in 43.7 % of scans and within 2 m in 20.9 % of scans. Social network analysis of 2 months of focal data revealed different structures for each of the ~~bipartite~~ proximity networks (Table 1). The social network visualisation of the 2-m scans partitioned the network into two large subgroups (parties), while visualisation of the 5-m scans appeared relatively cohesive and included all individuals at the level of the gang (Fig. 1). The 5-m network contained more individuals, more dyads, had a higher degree and a higher density than the 2-m network (Fig. 1; Table 1). Comparisons of degree centrality values between primary and secondary males revealed that for the 5-m network, secondary males had significantly higher degree centrality than primary males (median degree centrality for seven primary males = 3.0; median degree centrality for six secondary males = 3.5; $W = 10.5$, $P < 0.05$). However, this relationship was untestable for the 2-m network due to the small number of secondary males observed ($N = 3$) in comparison to primary males ($N = 6$). The modularity values indicated less substructuring between the subgroups identified in the 5-m network compared to the 2-m network (Fig. 1; Table 1). Both community detection measures identified comparable numbers of subgroups within the two networks, although the assignment of the individuals to subgroups varied slightly. In the 2-m network, each female was assigned to her primary male, the male with whom she had the strongest tie. Each 2-m subgroup consisted of 1 primary male, 0–2 secondary males and 1–4 adult females. For the 5-m network, community assignment algorithms failed with only 33.3 % of females being assigned to the same primary males as has been identified in the 2-m network.

Table 1

10.1007/s00265-015-2050-6

Weighted network size and metrics based on 5 m and 2 m proximity scans between intersexual dyads

Variables	~~Proximity~~Network	
	5 m	2 m
Total no. individuals	28	24
Total no. dyads	91	48
Degree range	1–12	1–7
Degree mean	6.5	4
Density	0.24	0.17
Modularity–spin glass	0.03	0.07
Total no. of subgroups–spin glass	6	6
Modularity–walktrap	0.07	0.56
Total no. of subgroups–walktrap	5	7

Fig. 1

Two weighted association networks calculated from scan sampling of female-male dyads at two different distances: **a** 5 m ($N = 28$ individuals, 91 dyads) and **b** 2 m ($N = 24$ individuals, 48 dyads). Data were aggregated over a 2-month period of stability. The *nodes* identify sex and status categories: females, *red circles* ; primary males, *blue squares*; secondary males, *green squares*. The width of the edges connecting female-male dyads indicates the frequency at which a dyad was observed. The *numbers* in each node indicate the community to which that node was assigned based on spin glass community identification

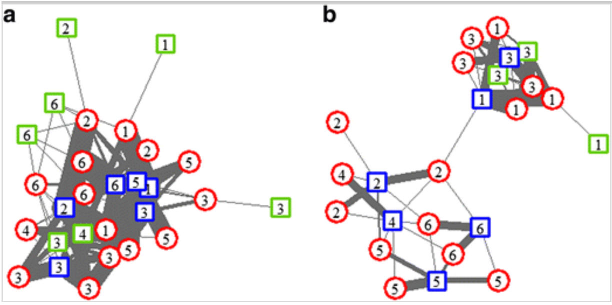

Similar results were obtained for the global assessment of ~10 months of focal data. We found a significantly preferred associate only for 2 of 16 females when we assessed the 5-m scans, while we identified preferred associates for 13 of 16 females when we assessed the 2-m scans (Friedman and Nemenyi tests; a subset of the results are available in Fig. S1).

Intersexual social behaviour and male social partner status

During focal sampling, grooming bout length varied between 0.07 and 23.15 min with a mean bout length of 3.51 min. In 76 % of total grooming time observed, females were actively grooming males. On average, females groomed with primary males 1.26 min/h of focal observation time and with secondary males 0.16 min/h. A typical grooming bout lasted on average 3.52 min ~~(± 0.10 min)~~ with primary males and 2.85 min ~~(± 0.08 min)~~ with secondary males. Females groomed significantly more frequently with primary than with secondary and unaffiliated males ($\chi^2 = 29.87$, $df = 2$, $P < 0.001$; Fig. 2a , Fig. S2). Greeting events occurred at a rate of 0.85 per hour. Ninety percent of greetings occurred between females and primary males, and greeting probability was significantly influenced by male status with females greeting significantly more with primary than with secondary and unaffiliated males ($\chi^2 = 39.27$, $df = 2$, $P < 0.001$; Fig. 2b). Aggressive behaviours, occurring at a rate of 0.10 events per hour (mean per female, ranging from 0 to 0.27 events per hour), customarily involved males behaving aggressively towards females; however, in 20 % of bouts, females were also observed to act aggressively towards males. Aggressive interactions occurred significantly more with primary than with secondary and unaffiliated males ($\chi^2 = 38.22$, $df = 2$, $P < 0.001$; Fig. 2c). Ad libitum data indicate that females in all reproductive states either actively or passively participated in aggressive behaviour with males and some counter-aggressive behaviours involved female-female coalitions. Primary males were responsible for 59 % of all infant-handling events by males with infants of focal females. Male status predicted the probability of infant-handling events, with primary males handling infants significantly more than secondary and unaffiliated males ($\chi^2 = 13.46$, $df = 2$, $P < 0.001$; Fig. 2d).

Fig. 2

The mean probabilities of observing **a** grooming, **b** greeting, **c** aggression and **d** infant handling between females and males of different status categories. *Horizontal black lines* show the models' predicted values. *Circles* represent the proportion of focal observations in which the respective behaviour was observed. The circle area is proportional to the number of observations and each female is represented by a *different colour*

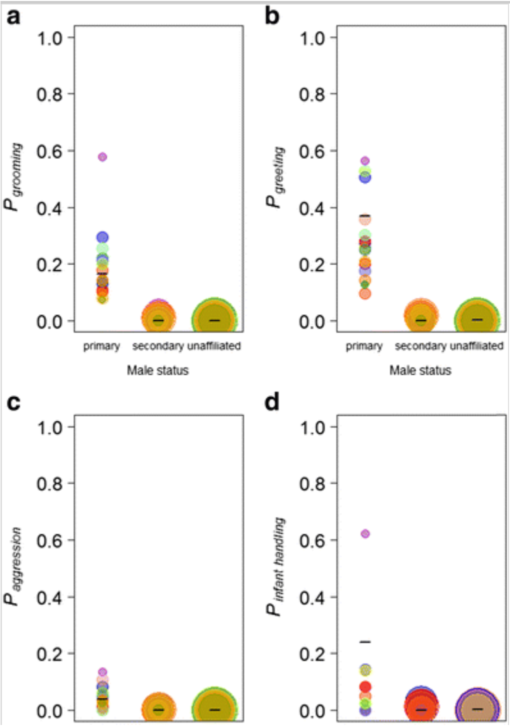

10.1007/s00265-015-2050-6

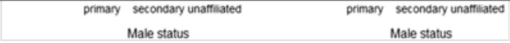

From six females, we were able to collect focal observations when they were tumescent. These females copulated with a total of 7 different males, 6 of which were adult and 1 subadult. Two females copulated with 1 male only, 4 females with 2 or more males, but were consistent with copulation partner within any respective oestrus period. For these six tumescent females, copulations occurred at a mean rate of 0.69 times per focal hour. The small sample size did not allow for assessing if male status influenced the number of copulations in the same manner presented above; therefore, we looked at the total number of copulations observed throughout the study period. Of 493 copulations observed ad libitum between 11 tumescent females and 12 males (10 adults and 2 subadults), 98.6 % occurred between females and their respective primary male.

On average, 4.9 secondary males (range = 0 to 10) were assigned to each female based on 2 m proximities. Yet again, proximity did not necessarily imply social interaction as females typically interacted with far fewer secondary males (e.g. mean number of secondary male grooming partners = 0.52; range = 0 to 3).

Directionality of relationship maintenance

Primary males were responsible for 60 % of all approaches (25 dyads), while secondary males initiated 76 % of all approaches (33 dyads). The HI ranged from –0.66 to 0.26 (mean = –0.17) for intersexual dyads containing primary males and –0.88 to 0.07 (mean = –0.36) for those with secondary males, indicating that in the majority of dyads, males were responsible for maintaining proximity to females (in 18 of 24 dyads containing primary males and 15 of 16 dyads containing secondary males; Fig. 3). No difference was found in the HI scores for dyads containing primary males vs. secondary males ($\chi^2 = 1.19$, $df = 1$, $P = 0.28$).

Fig. 3

The Hinde indices for intersexual dyads in which at least ten approach-retreat interactions were observed over the course of the study period. The *blue filled diamonds* represent dyads containing females and primary males and the *green open diamonds* represent dyads containing females and secondary males;. gGroup means for male status categories are indicated inby the *blue* (primary males) and *green* (secondary males) *horizontal lines*. The *black dashed line* indicates 0, where the responsibility for relationship maintenance is equal between females and males

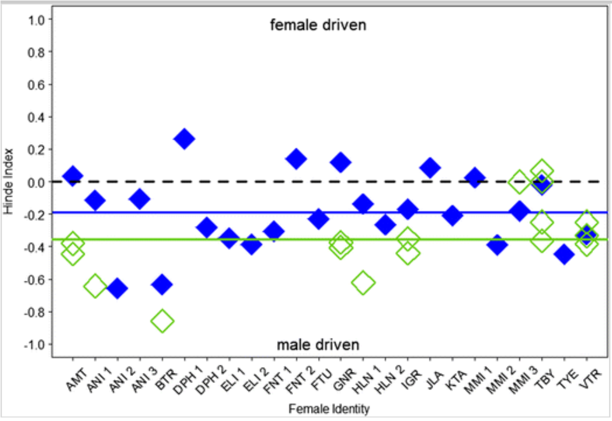

Female reproductive state and intersexual relationships

GLMMs of the probability of observing specific behaviours indicated that female reproductive state only minimally impacted the probability of social behaviours with primary males. The grooming probability ($\chi^2 = 7.98$, $df = 5$, $P = 0.16$) and aggression with primary males ($\chi^2 = 8.18$, $df = 5$, $P = 0.15$) did not vary significantly in relation to female reproductive state. However, female reproductive state significantly influenced greeting probability ($\chi^2 = 16.10$, $df = 5$, $P < 0.01$; Fig. 4). Post hoc analyses indicated that lactating females greeted with primary males significantly less often, while there was no difference between pregnant and cycling females (Table 2). The analysis of the duration of grooming bouts between females and primary males revealed no relationship between female reproductive state and grooming ($\chi^2 = 6.69$, $df = 5$, $P = 0.25$); female reproductive state also did not influence the frequency of greeting events ($\chi^2 = 2.96$, $df = 5$, $P = 0.71$).

Fig. 4

The mean probabilities of observing females greeting with their primary males in relation to the females' reproductive states. *Horizontal black lines* show the models' predicted values. *Circles* represent the proportion of focal observations in which greetings were observed. The circle area is proportional to the number of observations and each female is represented by a *different colour*. Female reproductive state categories: *L* lactating, *P* pregnant, *C0* cycling but detumescent, *C1* tumescent size 1 (small), *C2* tumescent size 2 (medium) and *C3* tumescent size 3 (large)

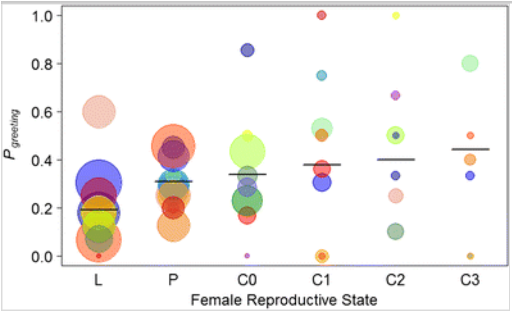

Table 2

The effects of reproductive state on the occurrence of greeting probability

Q4

	Estimate	Standard error	*z* value	*P* value
Intercept	–1.442	0.182	–7.907	<0.001
Pregnant	0.641	0.207	3.102	0.002
Detumescent C0	0.770	0.292	2.641	0.008
Tumescent C1	0.941	0.312	3.018	0.003
Tumescent C2	1.034	0.385	2.686	0.007
Tumescent C3	1.209	0.496	2.437	0.015

Results from a generalized linear mixed model with binomial error structure, in which female and male identity were included as random factors. The *intercept* represents lactating females

Seven of the focal females, in various reproductive states, maintained affiliative relationships (via grooming interactions) with males who were not their primary male (Fig. S2). Of the 36 grooming bouts with secondary males observed during focal observations, 61 % (from 13 of the 14 dyads) were non-surreptitious and females never received aggression from their primary males, although it was apparent that the primary males were aware of these interactions (either because they were participants (42 %), or were seated within 10 m and in a direct line of sight (19 %)). Some females were observed to share the same secondary males. Unfortunately, these interactions are too few to determine if secondary males are more likely to engage in social interactions with oestrus females.

10.1007/s00265-015-2050-6

An analysis of the influence female reproductive state on relationships with secondary males was not possible due to the small sample size. Ad libitum data indicate that females in all reproductive states were observed to groom and greet with secondary males, however.

Temporal dynamics of female-male associations

The use of ad libitum data allowed us to look at the changes in the identity of the primary males over a longer period than was possible with only focal data (Fig. 5). Changes in female-primary male affiliation, based on the occurrence of grooming, greeting and copulations, were immediately obvious, and females were observed to transfer between and within parties (intra-party transfers = 10, inter-party intra-gang transfers = 6, inter-gang = 2–4 (2 inter-gang transfers may have been unconfirmed mortalities)). The 16 females were distributed unevenly over 10 primary males in the focal gang, and the majority of females shared their primary male with at least one other female, and as many as four adult females sharing the same male. Females were not observed to transfer to their secondary males, but rather to bachelors or already established primary males. Although the exact moments of transfer were not observed during focal observations, there appears to be no graded period when females transferred from one primary male to the other. On two occasions, within a few hours or days of a transfer, social interactions between the new pair appeared to result in aggressive displays (i.e. stares and ground slaps) by the former primary male. In addition, during ad libitum data collection, dyadic male-male aggression was observed between a primary male and secondary male immediately following surreptitious affiliation. Seventeen changes were identified for females in various reproductive states (lactating, pregnant and cycling), and no infanticide was observed. Over the 507 study days, eight females remained with the same primary male, while eight females changed primary males at least once (Fig. 5). Changes in primary males occurred for females who did and did not have secondary male social partners at the time of transfer. As the exact moment that these transfers occurred was not observed, it is unclear as to whether males or females were the instigators. Female tenure time with any single male varied from 15 to 507 days (the complete observation period; Fig. 5). Median female tenure length was 200 days. However, this value may be a conservative estimate, as only 6 of 31 female tenures were not truncated by the study period (Figs. 5 and 6). Females interacted with secondary males at a much lower rate than with primary males, thus making shifts in secondary male status more difficult to detect. Four females maintained social relationships with secondary males for periods of >300 days.

Fig. 5

A schematic of the temporal changes in female associations with primary males. The identity of primary males is shown on the *y*-axis, with study females grouped on the inner *y*-axis by unit membership (as indicated by the *three-letter IDs* of the primary males). The *coloured lines* represent different females with *horizontal lines* showing persistent unit membership and *vertical lines* showing transfer between males. Study day is indicated on the *x*-axis. *Black horizontal lines* distinguish between parties with consistent membership (separated by a *solid line*) and parties with males who changed their affiliation (separated by *dashed lines*): party IDs 4, 9 and 10

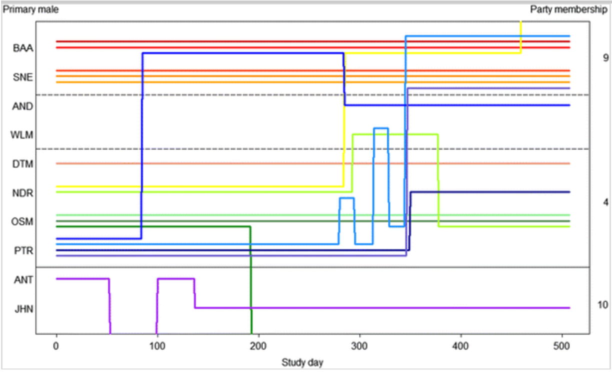

Fig. 6

Histogram of female tenure length from ad libitum data. *Grey bars* indicate tenures which were truncated due to the study duration (507 study days), and *white bars* indicate the tenures for which the start and the end were observed

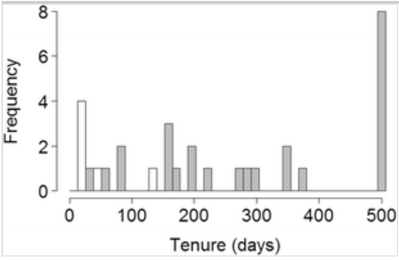

Discussion

The primary aim of this study was to provide comprehensive data on female-male relationships in wild Guinea baboons, to fill in the gaps in our understanding of this species' social system and, ultimately, to contribute to a better understanding of primate social evolution. The social network analysis not only corroborated the existence of parties within gangs (Patzelt et al. 2014), but also identified further substructures ("units") within parties, which comprised 1–3 adult and subadult males and 1–4 adult females. These units became only apparent when close spatial associations (up to 2 m), but not medium distances (up to 5 m) were considered. Within units, females showed strong spatial associations with one specific primary male, and most of the social interactions were confined to that male. Some females groomed with other secondary males. These males were typically subadult, post-prime or injured males. Some relationships with secondary males lasted throughout the study period.

Female reproductive state only marginally affected the frequency and type of interactions with primary males. The most striking finding was that although females spent substantial amounts of time outside a distance of 5 m from any male, mate fidelity was remarkably high, as almost all of the observed copulations were restricted to the primary male. Thus, from the females' perspective, the mating system seems to be monandrous. Given these mating patterns and the social and spatial relationships between females and their respective primary male "OMUs" appear to comprise the core of the Guinea baboon society (Table 3), confirming earlier observations on mating behaviour in captivity (Boese 1973 ; Maestripieri et al. 2005, ~~; Maestripieri et al.~~ 2007). At the level of the social organisation, some of the subunits constitute multi-male units, as there may be one o~~f~~r more secondary males (Kummer 1968 ; Dunbar and Dunbar 1975 ; Dunbar 1984 ; Pines et al. 2011 ; Snyder-Mackler et al. 2012a ; Chowdhury et al. 2015). Multiple units are embedded within the party and two or more parties come together to form a gang. Gangs may be comparable to the bands of hamadryas baboons and geladas or troops in savannah baboons (c.f. Dunbar 1984).

Table 3

Features of intersexual relationships in *Papio* and *Theropithecus*

Q5

Spatial and behavioural features	MM MF	OMUs		This study
	~~S~~savannah baboons	~~H~~hamadryas baboon	Gelada	Guinea baboon
FRS predicts affiliation	Strong[a]	Weak[b]	Weak[c]	Weak
Distance to male: L~~/~~ or P	n/a	Mean = 2.2 ± 1.5 m[d]	13.0 ± 4.0 % at 2 m[e]	18.8 ± 1 % at 2 m
Approaches by males	n/a	28 %[f]	Male driven[e]	76 %
Grooming	n/a	*L* > *F* [d]	Differentiated[c,g]	*L* > *S*
Aggression/herding (hourly rate)	n/a	>0.25[h]	0.23[c]	0.1
Female counter-aggression and coalitions	n/a	Absent[i]	Present[c,g]	Present
Female transfer	n/a	Individual[b,j–l]	Group[c]	Individual
Range of OMU sizes	n/a	1–9[d]	1–10[c]	1–4

10.1007/s00265-015-2050-6

Mean OMU size	n/a	2.6[l]	5.07–6.25[m]	2.14

n/a not applicable, *L* leader, *F* follower, *P* primary, *S* secondary, *FRS* female reproductive state

[a](Seyfarth and Cheney 2012)

AQ6

[b](Kummer 1968)

[c](Dunbar and Dunbar 1975)

[d](Swedell 2006)

[e](Kawai and Mori 1979)

[f](Kummer 1990)

[g](Dunbar 1984)

[h](Swedell and Schreier 2009)

[i](Swedell 2011)

[j](Sigg et al. 1982)

[k](Swedell 2000)

[l](Swedell et al. 2011)

[m](Snyder-Mackler et al. 2012a)

The comparative perspective

Intersexual relationships in Guinea baboons share some interesting similarities with hamadryas baboons (Table 3). Both species have superficially similar nested multilevel systems containing OMUs, but there are also marked differences (Table 3). Notably, in hamadryas baboons, males enforce close female proximity through herding (Kummer 1968 ; Swedell and Schreier 2009), in a similar fashion as in other harem-based societies, such as horses (*Equus ferus caballus*, Monard and Duncan 1996). Hamadryas baboon females submit to male coercion through early conditioning and futility of opposition, and thereby learn to maintain close spatial proximity to their leader male (Kummer 1990 ; Swedell and Schreier 2009), but it may also be in the female's best interest to stay in the proximity of a particular male. Takeovers of adult females in hamadryas baboons often involve male-male conflicts and are the result of the defeat of an older leader male. During male takeovers, OMUs are frequently split up, with females of the original OMU found in different OMUs afterwards (Kummer 1968 ; Sigg et al. 1982 ; Swedell 2000 ; Swedell et al. 2011).

Guinea baboon females spend substantial amounts of time away from any male, implying that females have a certain degree of freedom not available to hamadryas females. Interestingly, Guinea baboon females respond to male aggression with occasional counter-aggression and female-female coalitions, rather than the submissive behaviour characteristic of hamadryas females. Females take an active role in relationship continuity and are seemingly able to avoid advances by other males. In Guinea baboons, transfers of females between different primary males occurred individually. The level of the social system did not halt female transfers as females were observed to change between OMUs at all three social levels. Interestingly, sometimes, the transfer of one female was shortly followed by the transfer of other females, resulting in periods of social instability (see Fig. 5 ; Table 3). This raises the question to which degree females compete over males, an aspect that has previously often been neglected (Clutton-Brock and Huchard 2013), although Kummer (1968) reported frequent female-female competition for access to the leader male in hamadryas baboons.

The multilevel social system of geladas (Mori 1979 ; Dunbar 1984) offers an alternative female-bonded social pattern, which has some similarities to Guinea baboons in that female counter-aggression and coalition formation have been observed (Table 3). In contrast, gelada unit cohesion is explained by strong female kin-based relationships (le Roux et al. 2011). Gelada OMUs appear to be larger, are less spatially separated and may overlap with other OMUs (Kawai and Mori 1979 ; Snyder-Mackler et al. 2012b). Further substructuring may be caused by the splitting/budding of OMUs as their size increases (Dunbar 1984). Males commonly acquire females through taking over a group of closely related females (Dunbar and Dunbar 1975 ; Dunbar 1984), and occasionally, followers may lure females from an OMU ~~from~~with which they are affiliated (Dunbar 1984).

In sum, we conclude that female-male relationships in Guinea baboons differ fundamentally from those of savannah baboons, where females maintain close associations with males only during consortships, and with male "friends" when they are lactating, while they share greater similarities with those between hamadryas baboon males and females.

Female and male reproductive strategies

Male competition for access to mates (Dobson 1982) and control over females var~~y~~ies substantially between species (Smuts and Smuts 1993). In a number of multilevel species, prime males at their reproductive peak actively exclude male competitors and sequester females (Rubenstein 1994 ; Linklater 2000 ; le Roux and Bergman 2012 ; Qi et al. 2014 ; Chowdhury et al. 2015). In cases where complete exclusion of outside males is not possible, dominant-prime males concede to the presence of other males who may assist in territorial or female defence (Kummer 1968 ; Mori 1979 ; Linklater et al. 1999 ; Rubenstein and Hack 2004). This may prolong leader male tenure, but may also result in reproductive concessions (Feh 1999 ; Snyder-Mackler et al. 2012a ; Chowdhury et al. 2015). Yet males may not concede, but rather cooperate to increase their reproductive benefit, such as in bottlenose dolphins (*Tursiops aduncus:* Wiszniewski et al. 2012).

High mate fidelity between Guinea baboon females and primary males indicates that primary males are not making reproductive concessions to other males, although paternity data will be needed to corroborate this assumption. The high degree of mate fidelity, the low overt competition by males for mating opportunities (Kalbitzer et al. 2015) and the fact that Guinea baboon males show relatively small testes (Patzelt 2013) are consistent with a monogamous or polygyny-monandrous mating system where sperm competition does not play a major role (Jolly and Phillips-Conroy 2003 , 2006).

The adaptive value of friendships between females and secondary males in Guinea baboons presently remains unclear. Furthermore, our results raise the question why Guinea baboon males make hardly any overt attempts to control or takeover females from other males. One conjecture is that males forego competition over females because this might jeopardise their bonds with other males (Patzelt et al. 2014). The occurrence of closely related males within the party (Patzelt et al. 2014) may alleviate some of the costs associated with lost reproductive opportunities. Long-term data will be needed to assess the roles that females and males play in maintaining long-term relationships and the predictors of female transfers between males, to obtain a full understanding of female and male strategies.

Evolution of social systems

One major debate in the understanding of social evolution is the interplay between phylogenetic inertia (as outcomes of past selective pressures and genetic drift) and current ecological conditions. The standard socio-ecological model predicts that male mammals map themselves onto female distribution patterns, which are driven by resource distribution (Jarman 1974 ; Emlen and Oring 1977 ; Clutton-Brock 1989 ; Sterck et al. 1997). Grueter and van Schaik (2009) proposed that multilevel groups are better equipped to balance the costs and benefits of group living, which may not only apply to nonhuman primates but also to some wild equids (Rubenstein 1986 , 1994 ; Rubenstein et al. 2007), African and Asian elephants (de Silva et al. 2011 ; de Silva and Wittemyer 2012), certain antelope species (Jarman 1974) and perhaps giraffes (VanderWaal et al. 2014).

According to phylogenetic reconstructions, the ancestral state of the social system in ~~p~~Papionins was most likely a female-bonded multi-male multi-female system (Di Fiore and Rendall 1994). The multilevel system found in hamadryas, Guinea baboons and geladas thus represents a derived trait. Since geladas and baboons have a relatively long independent evolutionary history (Delson 1993 ; Newman et al. 2004 ; Liedigk et al. 2014), it can be assumed that the multilevel systems of geladas and baboons evolved independently (Grueter et al. 2012). In contrast, hamadryas and Guinea baboons have a common ancestor which lived less than 2 ~~Ma~~million years ago (Zinner et al. 2009 ; Liedigk et al. 2014), indicating that the OMU-based multilevel system of these species may be a synapomorphic trait already present in their last common ancestor. Jolly (2009) proposed that spatial dynamics during that range expansion may have played a role in shaping baboon social systems. Conditions at the frontier of the range expansion might have favoured male philopatry and promoted a shift from the female-bonded to a male-bonded system (Jolly 2009). The frontier population(s) constituted the ancestors of extant hamadryas and Guinea baboons.

While this scenario stresses the ecological and demographic conditions in the past, others have focussed on present-day ecological conditions. Specifically, the social organisation of hamadryas baboons and geladas has been viewed as adaptations to extreme and somewhat marginal habitats (Dunbar 1992 ; Schreier and Swedell 2012). However, the ecology of the two species differs greatly and therefore, a simple relationship between particular ecological settings (i.e. spatial and temporal distribution of resources) and the respective social system cannot be inferred.

AQ7

Taken together, there is still no single comprehensive model that integrates phylogenetic descent with present-day factors. Resource availability, predation pressure, infanticide risk and bachelor threat may all have potentially affected the social dynamics and social evolution of the different variants of multilevel societies (Rubenstein 1986 ; Grüter and Zinner 2004 ; Grueter et al. 2012). We suggest that fundamental characteristics in social tendencies (e.g. aggressiveness and mating pattern) indeed have a genetic basis, while present-day ecological conditions drive short-term variation in social organisation (Sharman 1982). Hybrid zones may prove useful to investigate this natural interplay between these two factors. Behavioural studies of hamadryas-olive baboon hybrid groups (Sugawara 1979 ; Müller 1980; Beehner 2003 ; Bergman and Beehner 2004) have already indicated that there may be a genetic basis to male herding behaviour. Future work combining behavioural and genetic studies on Guinea-olive baboon hybrids would contribute to our understanding of the genetic basis of male physical coercion of females as well as the extent to which females can and do exhibit choice.

AQ8

Acknowledgments

10.1007/s00265-015-2050-6

We would like to thank the Diréction des Parcs Nationaux (DPN) and Ministère de l'Environnement et de la Protéction de la Nature (MEPN) de la République du Sénégal for permission to work in the Park National de Niokolo Koba (PNNK), as well as the conservateur of PNNK, Mamadou Sidibé, for his logistical support. We have much appreciation for the staff and researchers of the Centre de Recherche de Primatologie Simenti, in particular Cheikh Sané, Moustapha Faye, Armél Louis Nyafouna and Ibrahima Diatta for their companionship and Federica Dal Pesco, Ulrike Barnett and Matthias Klapproth for their assistance in the field. We also thank Roger Mundry and Holger Sennhenn-Reulen for providing statistical aid and Robin Dunbar, Dorothy Cheney, Cédric Girard-Buttoz, Gisela Kopp and three anonymous reviewers for commenting on earlier versions of this manuscript. Funding from the Deutsche Forschungsgemeinschaft (DFG Fi707/9) is gratefully acknowledged.

Compliance with ethical standards

Approval and research permission was granted by the ~~Diréction des Parcs Nationaux (~~DPN~~)~~ and the ~~Ministère de l'Environnement et de la Protéction de la Nature (~~MEPN~~)~~ de la République du Sénégal (research permit numbers: 0383/24/03/2009; 0373/10/3/2012). Research was conducted within the regulations set by Senegalese agencies as well as by the Animal Care Committee at the German Primate Center.

Conflict of interest The authors declare that they have no conflict of interest.

Ethical approval All applicable international, national, and/or institutional guidelines for the care and use of animals were followed. All procedures performed in studies involving animals were in accordance with the ethical standards of the institution or practice at which the studies were conducted.

Electronic supplementary material

Below is the link to the electronic supplementary material.

ESM 1

(DOCX 192 kb)

References

Altmann J (1974) Observational study of behavior: sampling methods. Behaviour 49:227–267

Baayen RH, Davidson DJ, Bates DM (2008) Mixed-effects modeling with crossed random effects for subjects and items. J Mem Lang 59:390–412

Bates D, Maechler M, Bolker BM, Walker S (2013) lme4: linear mixed-effects models using Eigen and S4, http://CRAN.R-project.org/package=lme4

Beehner JC (2003) Female behavior and reproductive success in a hybrid baboon group. Doctoral dissertation, Washington University

Begg CM, Begg KS, Du Toit JT, Mills MGL (2006) Spatial organization of the honey badger *Mellivora capensis* in the southern Kalahari: home-range size and movement patterns. J Zool 265:23–35

Bergman TJ, Beehner JC (2004) Social system of a hybrid baboon group (*Papio anubis* X *P. hamadryas*). Int J Primatol 25:1313–1330

Boese G (1973) Behavior and social organization of the Guinea baboon (*Papio papio*). Doctoral dissertation, The Johns Hopkins University

Castles M, Heinsohn R, Marshall HH, Lee AEG, Cowlishaw G, Carter AJ (2014) Social networks created with different techniques are not comparable. Anim Behav 96:59–67

Chowdhury S, Pines M, Saunders J, Swedell L (2015) The adaptive value of secondary males in the polygynous multi-level society of hamadryas baboons: adaptive value of secondary males in a polygynous primate. Am J Phys Anthropol 158:501–513

Clutton-Brock TH (1989) Mammalian mating systems. Proc R Soc Lond B 236:339–372

Clutton-Brock T, Huchard E (2013) Social competition and its consequences in female mammals: female reproductive competition in mammals. J Zool 289:151–171

R Core Team (2014) R: ~~A~~a language and environment for statistical computing. R Foundation for Statistical Computing, Vienna, Austria, http://www.r-project.org

Csárdi G, Nepusz T (2006) The igraph software package for complex network research. InterJournal Complex Syst 1695:1–9

Davies NB, Lundberg A (1984) Food distribution and a variable mating system in the Dunnock, *Prunella modularis*. J Anim Ecol 53:895–912

de Silva S, Wittemyer G (2012) A comparison of social organization in Asian elephants and African savannah elephants. Int J Primatol 33:1125–1141

de Silva S, Ranjeewa A, Kryazhimskiy S (2011) The dynamics of social networks among female Asian elephants. BMC Ecol 11:17

Delson E (1993) *Theropithecus* fossils from Africa and India and the taxonomy of the genus. In: Jablonski NG (ed) *Theropithecus*: The rise and fall of a primate genus. Cambridge University Press, Cambridge, pp 157–189

Demšar J (2006) Statistical comparisons of classifiers over multiple data sets. J Mach Learn Res 7:1–30

Di Fiore A, Rendall D (1994) Evolution of social organization: a reappraisal for primates by using phylogenetic methods. Proc Natl Acad Sci U S A 91:9941–9945

Dobson FS (1982) Competition for mates and predominant juvenile male dispersal in mammals. Anim Behav 30:1183–1192

Dunbar RIM (1984) Reproductive decisions: an economic analysis of gelada baboon social strategies. Princeton University Press, Princeton, New Jersey

Dunbar RIM (1992) A model of the gelada socio-ecological system. Primates 33:69–83

Dunbar RIM, Dunbar P (1975) Social dynamics of gelada baboons. S. Karger, Basel

Dunbar RIM, Nathan MF (1972) Social organization of the Guinea baboon, *Papio papio*. Folia Primatol 17:321–334

Emlen ST, Oring LW (1977) Ecology, sexual selection, and the evolution of mating systems. Science 197:215–223

Feh C (1999) Alliances and reproductive success in Camargue stallions. Anim Behav 57:705–713

Fernando P, Lande R (2000) Molecular genetic and behavioral analysis of social organization in the Asian elephant (*Elephas maximus*). Behav Ecol Sociobiol 48:84–91

Field AP (2009) Discovering statistics using SPSS, 3rd edn. SAGE, Los Angeles

Friedman M (1940) A comparison of alternative tests of significance for the problem of m rankings. Ann Math Stat 11:86–92

Fruchterman TM, Reingold EM (1991) Graph drawing by force-directed placement. Softw Pract Exp 21:1129–1164

Galat-Luong A, Galat G, Hagell S (2006) The social and ecological flexibility of Guinea baboons: implications for Guinea baboon social organization and male strategies. In: Swedell L, Leigh SR (eds) Reproduction and fitness in baboons: behavioral, ecological, and life history perspectives. Springer, New York, pp 105–121

Gauthier C-A (1999) Reproductive parameters and paracallosal skin color changes in captive female Guinea baboons, *Papio papio*. Am J Primatol 47:67–74

Greenwood PJ (1980) Mating systems, philopatry and dispersal in birds and mammals. Anim Behav 28:1140–1162

Grueter CC, van Schaik CP (2009) Evolutionary determinants of modular societies in colobines. Behav Ecol 21:63–71

Grueter CC, Chapais B, Zinner D (2012) Evolution of multilevel social systems in nonhuman primates and humans. Int J Primatol 33:1002–1037

Grüter CC, Zinner D (2004) Nested societies: convergent adaptation of baboons and snub-nosed monkeys? Primate Rep 70:1–98

Higham JP, Semple S, MacLarnon A, Heistermann M, Ross C (2009) Female reproductive signaling, and male mating behavior, in the olive baboon. Horm Behav 55:60–67

Hinde RA (1977) On assessing the bases of partner preferences. Behaviour 62:1–9

Hinde RA, Atkinson S (1970) Assessing the roles of social partners in maintaining mutual proximity, as exemplified by mother-infant relations in rhesus monkeys. Anim Behav 18:169–176

Jarman P (1974) The social organisation of antelope in relation to their ecology. Behaviour 48:215–267

Jolly CJ (2009) Fifty years of looking at human evolution: backward, forward, and sideways. Curr Anthropol 50:187–199

Jolly CJ, Phillips-Conroy JE (2003) Testicular size, mating system, and maturation schedules in wild anubis and hamadryas baboons. Int J Primatol 24:125–142

Jolly CJ, Phillips-Conroy JE (2006) Testicular size, developmental trajectories, and male life history strategies in four baboon taxa. In: Swedell L, Leigh SR (eds) Reproduction and fitness in baboons: behavioral, ecological, and life history perspectives. Springer, New York, pp 257–275

Kalbitzer U, Heistermann M, Cheney D, Seyfarth R, Fischer J (2015) Variation in aggressiveness and spatial tolerance between male chacma and Guinea baboons in relation to testosterone and glucocorticoid levels. Horm Behav 75:100–110

Kawai M, Mori U (1979) Spacing within units and unit integrity. In: Kawai M (ed) Ecological and sociological studies of gelada baboons. Karger, Basel, pp 199–217

Kopp GH, Fischer J, Patzelt A, Roos C, Zinner D (2015) Population Genetic Insights into the Social Organization of Guinea Baboons (*Papio papio*): Evidence for Female-Biased Dispersal. Am J Primatol. doi: 654 10.1002/ajp.22415

Kreft I, Dde Leeuw J (1998) Introducing multilevel modeling. Sage, London

Kummer H (1968) Social organization of hamadryas baboons, a field study. University of Chicago Press, Chicago

Kummer H (1990) The social system of Hamadryas baboons and its presumable evolution. In: Thiago de Mello M, Whiten A, Byrne RW (eds) Baboons: behaviour and ecology. Use and care. Selected Proceedings of the XIIth Congress of the International Primatological Society. Brasilia, Brasil., pp 43–60

le Roux A, Bergman TJ (2012) Indirect rival assessment in a social primate, *Theropithecus gelada*. Anim Behav 83:249–255

le Roux A, Beehner JC, Bergman TJ (2011) Female philopatry and dominance patterns in wild geladas. Am J Primatol 73:422–430

Lemasson A, Palombit RA, Jubin R (2008) Friendships between males and lactating females in a free-ranging group of olive baboons (*Papio hamadryas anubis*): evidence from playback experiments. Behav Ecol Sociobiol 62:1027–1035

Liedigk R, Roos C, Brameier M, Zinner D (2014) Mitogenomics of the Old World monkey tribe Papionini. BMC Evol Biol 14:176

Linklater WL (2000) Adaptive explanation in socio-ecology: lessons from the Equidae. Biol Rev Camb Philos Soc 75:1–20

Linklater WL, Cameron EZ, Minot EO, Stafford KJ (1999) Stallion harassment and the mating system of horses. Anim Behav 58:295–306

Lusseau D (2003) The emergent properties of a dolphin social network. Proc R Soc Lond B 270:S186–S188

Maciej P, Patzelt A, Ndao I, Hammerschmidt K, Fischer J (2012) Social monitoring in a multilevel society: a playback study with male Guinea baboons. Behav Ecol Sociobiol 67:61–68

Maestripieri D, Leoni M, Raza SS, Hirsch EJ, Whitham JC (2005) Female copulation calls in Guinea baboons: evidence for postcopulatory female choice? Int J Primatol 26:737–758

Maestripieri D, Mayhew J, Carlson CL, Hoffman CL, Radtke JM (2007) One-male harems and female social dynamics in Guinea baboons. Folia Primatol 78:56–68

Mitani JC (1990) Experimental field studies of Asian ape social systems. Int J Primatol 11:103–126

Molnar PK, Derocher AE, Lewis MA, Taylor MK (2008) Modelling the mating system of polar bears: a mechanistic approach to the Allee effect. Proc R Soc Lond B 275:217–226

Monard A-M, Duncan P (1996) Consequences of natal dispersal in female horses. Anim Behav 52:565–579

Mori U (1979) Inter-unit relationships. In: Kawai M (ed) Ecological and sociological studies of gelada baboons. Karger, Basel, pp 83–92

Muller MN, Kahlenberg SM, Thompson EM, Wrangham RW (2007) Male coercion and the costs of promiscuous mating for female chimpanzees. Proc R Soc Lond B 274:1009–1014

Newman TK, Jolly CJ, Rogers J (2004) Mitochondrial phylogeny and systematics of baboons (*Papio*). Am J Phys Anthropol 124:17–27

Palombit R (2009) “Friendship” with males: a female counterstrategy to infanticide in chacma baboons of the Okavango Delta. In: Muller MN, Wrangham RW (eds) Sexual coercion in primates and humans. Harvard University Press, Cambridge, MA, pp 377–409

Patzelt A (2013) The social system of Guinea baboons (*Papio papio*) with a focus on male-male relationships. Doctoral dissertation, Georg-August-Universität Göttingen

Patzelt A, Kopp GH, Ndao I, Kalbitzer U, Zinner D, Fischer J (2014) Male tolerance and male-male bonds in a multilevel primate society. Proc Natl Acad Sci U S A 111:14740–14745

Pines M, Saunders J, Swedell L (2011) Alternative routes to the leader male role in a multi-level society: follower vs. solitary male strategies and outcomes in hamadryas baboons. Am J Primatol 73:679–691

Pohlert T (2014) The pairwise multiple comparison of mean ranks package (PMCMR), http://CRAN.R-project.org/package=PMCMR

Qi X-G, Garber PA, Ji W et al (2014) Satellite telemetry and social modeling offer new insights into the origin of primate multilevel societies. Nat Commun 5:5296

Ridley MA (1986) The number of males in a primate troop. Anim Behav 34:1848–1858

Rubenstein DI (1986) Ecology and sociality in horses and zebras. In: Rubenstein DI, Wrangham RW (eds) Ecological aspects of social evolution. Princeton University Press, Princeton, New Jersey, pp 282–302

Rubenstein DI (1994) The ecology of female social behaviour in horses, zebras and asses. In: Jarman P, Rossiter A (eds) Animal societies: individuals, interactions, and organization. Kyoto University Press, Kyoto, Japan, pp 13–28

Rubenstein DI, Hack M (2004) Natural and sexual selection and the evolution of multi-level societies: insights from zebras with comparisons to primates. In: Kappeler PM, van Schaik C (eds) Sexual selection in primates. Cambridge University Press, pp 266–279

Rubenstein DI, Sundaresan S, Fischhoff I, Saltz D (2007) Social networks in wild asses: comparing patterns and processes among populations. In: Stubbe A, Kaczensky P, Wesche K, Samjaa R, Stubbe M (eds) Exploration into the biological resources of Mongolia, Martin-Luther-University Halle-Wittenberg, Halle (Saale), vol. 10, pp 159–176

Schreier AL, Swedell L (2009) The fourth level of social structure in a multi-level society: ecological and social functions of clans in hamadryas baboons. Am J Primatol 71:948–955 (2012) Ecology and sociality in a multilevel society: ecological determinants of spatial cohesion in hamadryas baboons. Am J Phys Anthropol 148:580–588

AQ9

Seyfarth RM, Cheney DL (2012) The Evolutionary Origins of Friendship. Annu Rev Psychol 63:153–177

Sharman MJ (1982) Feeding, ranging and social organisation of the Guinea baboon. Doctoral dissertation, University of St Andrews

Sigg H, Stolba A, Abegglen J-J, Dasser V (1982) Life history of hamadryas baboons: physical development, infant mortality, reproductive parameters and family relationships. Primates 23:473–487

Smuts BB, Smuts RW (1993) Male aggression and sexual coercion of females in nonhuman primates and other mammals: evidence and theoretical implications. Adv Stud Behav 22:1–63

Snyder-Mackler N, Alberts SC, Bergman TJ (2012a) Concessions of an alpha male? Cooperative defence and shared reproduction in multi-male primate groups. Proc R Soc Lond B 279:3788–3795

Snyder-Mackler N, Beehner JC, Bergman TJ (2012b) Defining higher levels in the multilevel societies of Geladas (*Theropithecus gelada*). Int J Primatol 33:1054–1068

Städele V, Van Doren V, Pines M, Swedell L, Vigilant L (2015) Fine-scale genetic assessment of sex-specific dispersal patterns in a multilevel primate society. J Hum Evol 78:103–113

Sterck EHM, Watts DP, van Schaik CP (1997) The evolution of female social relationships in nonhuman primates. Behav Ecol Sociobiol 41:291–309

R Studio (2012) RStudio: integrated development environment for R. RStudio Inc, Boston, Massachusetts

Sugawara K (1979) Sociological study of a wild group of hybrid baboons between *Papio anubis* and *P. hamadryas* in the Awash Valley, Ethiopia. Primates 20:21–56

Sundaresan SR, Fischhoff IR, Rubenstein D (2007) Male harassment influences female movements and associations in Grevy’s zebra (*Equus grevyi*). Behav Ecol 18:860–865

Swedell L (2000) Two takeovers in wild hamadryas baboons. Folia Primatol 71:169–172

Swedell L (2006) Strategies of sex and survival Hamadryas baboons: through a female lens. Pearson Prentice Hall, Upper Saddle River, New Jersey

Swedell L (2011) African ~~p~~Papionins: diversity of social organization and ecological flexibility. In: Campbell CJ, Fuentes A, MacKinnon KC, Panger M, Bearder SK (eds) Primates in perspective, 2nd edn. Oxford University Press, New York, pp 241–277

Swedell L, Schreier A (2009) Male aggression towards females in hamadryas baboons: conditioning, coercion, and control. In: Muller MN, Wrangham RW (eds) Sexual coercion in primates and humans: an evolutionary perspective on male aggression against females. Harvard University Press, Cambridge, pp 244–268

Swedell L, Saunders J, Schreier A, Davis B, Tesfaye T, Pines M (2011) Female “dispersal” in hamadryas baboons: transfer among social units in a multilevel society. Am J Phys Anthropol 145:360–370

Szykman M, Engh AL, Van Horn RC, Boydston EE, Scribner KT, Holekamp KE (2003) Rare male aggression directed toward females in a female-dominated society: baiting behavior in the spotted hyena. Aggress Behav 29:457–474

Szykman M, Van Horn RC, Engh AL, Boydston EE, Holekamp KE (2007) Courtship and mating in free-living spotted hyenas. Behaviour 144:815–846

VanderWaal KL, Wang H, McCowan B, Fushing H, Isbell LA (2014) Multilevel social organization and space use in reticulated giraffe *(Giraffa camelopardalis)*. Behav Ecol 25:17–26

Wiszniewski J, Corrigan S, Beheregaray LB, Möller LM (2012) Male reproductive success increases with alliance size in Indo-Pacific bottlenose dolphins (*Tursiops aduncus*): reproductive success in bottlenose dolphins. J Anim Ecol 81:423–431

Zinner D, Groeneveld L, Keller C, Roos C (2009) Mitochondrial phylogeography of baboons (*Papio* spp.)–indication for introgressive hybridization? BMC Evol Biol 9:83
