# Supplementary material for: Sex and friendship in a multilevel society: behavioural patterns and associations between female and male Guinea baboons
Source: Behav Ecol Sociobiol. 2016 Jan 22;70:323–36. doi: 10.1007/s00265-015-2050-6 (PMC4748025; doi:10.1007/s00265-015-2050-6)
Supplement: Supplementary file 1 — (DOCX 731 kb) [file 265_2015_2050_MOESM1_ESM.docx › upload_1/265_2015_2050_MOESM1_ESM_revised.docx]

Electronic Supplementary Material to complement an article published in

Behavioral Ecology and Sociobiology

**Sex and friendship in a multilevel society: behavioural patterns and associations between female and male Guinea baboons**

**Adeelia S. Goffe*, Dietmar Zinner & Julia Fischer**

Cognitive Ethology Laboratory, German Primate Center, 37077 Göttingen, Germany

* e-mail: agoffe@dpz.eu

**Supplementary Methods**

**Intersexual network structure**

Network density calculates the proportion of connections/edges between two nodes given the number of possible connections in a network and gives a global value of the interconnections within the network. Density values range from 0, relatively solitary individuals with no connections, to 1, a closely knit network where all individuals are equally connected to each other (Wasserman and Faust 1994). Traditionally, clustering (also called transitivity) is used to determine fine scale substructuring within a network. However, as our data contain only intersexual dyads it was not possible to base subgroup assignment on triadic connections. Therefore we used two other methods from community detection to identify subgroups within the two networks, as subgroup assignment based on a modularity maximising algorithm is not always biologically relevant (Kasper and Voelkl 2009). Spin glass, an approach from statistical physics based on the Potts model, optimises clustering through a process of simulated annealing based on the modularity of the network (Reichardt and Bornholdt 2006). The walktrap algorithm utilises random walks to identify subgroups in dense graphs (Pons and Latapy 2005). Both of these measures calculate modularity, or the reliability of the subdivisions of a network into smaller subgroups (Newman and Girvan 2004) and variations in modularity values are dependent on the splitting techniques used by the specific algorithms. As we wanted to consider the frequency at which males were present and unweighted edges may result in spurious results (Fagiolo et al. 2008), as well as lead to a reduction in the amount of data, we chose to use weighted edges in all analyses. Subgroup assignments of intersexual dyads derived from the spin glass (set at 500 spins) and walktrap algorithms were compared to the manual assignment of primary males described in the main text.

**Identification of male partners: Friedman test and Nemenyi test**

The Friedman average rank test (Friedman 1940) is a nonparametric repeated measures ANOVA which is performed by “ranking” different conditions in multiple observations and then determining the average rank (Friedman 1940; Demšar 2006; Field 2009). The null hypothesis is that all conditions are equivalent in their ranks. If the null hypothesis is rejected, that is the P-value is significant, then a Nemenyi test may be performed to identify which condition(s) differ (Demšar 2006). In R, the Nemenyi test reveals a matrix of P-values for all possible pairwise comparisons in each dataset. Here, for each female we treated the 20 males as the condition and the number of observations varied depending on how many scans were performed. Two data sets were run for each female (5 m proximity scans and 2 m proximity scans). We then rejected the null hypothesis in all cases as the Friedman test indicated significant values for all 32 tests (16 females, 2 dataset per female) and performed the Nemenyi postdoc analysis.

**References**

Demšar J (2006) Statistical comparisons of classifiers over multiple data sets. J Mach Learn Res 7:1–30

Fagiolo G, Reyes J, Schiavo S (2008) On the topological properties of the world trade web: a weighted network analysis. Phys A 387:3868–3873

Field AP (2009) Discovering statistics using SPSS, 3rd ed. SAGE, Los Angeles

Friedman M (1940) A comparison of alternative tests of significance for the problem of m rankings. Ann Math Stat 11:86–92

Kasper C, Voelkl B (2009) A social network analysis of primate groups. Primates 50:343–356

Newman MEJ, Girvan M (2004) Finding and evaluating community structure in networks. Phys Rev E 69:026113

Pons P, Latapy M (2005) Computing communities in large networks using random walks. In: Computer and Information Sciences-ISCIS 2005. Springer Berlin Heidelberg, pp 284–293

Reichardt J, Bornholdt S (2006) Statistical mechanics of community detection. Phys Rev E 74:016110

Wasserman S, Faust K (1994) Social Network Analysis: Methods and Applications. Cambridge University Press

**Supplementary Figures**

**Fig. S1** Relative occurrences of 20 adult and subadult males from **a** 5 m and **b** 2 m of four of the sixteen focal females. *Three letter codes* indicate individual baboons. *Asterisks* indicate males who were significantly more likely than others to be located within 5 m or 2 m of a given focal female according to the Nemenyi posthoc test


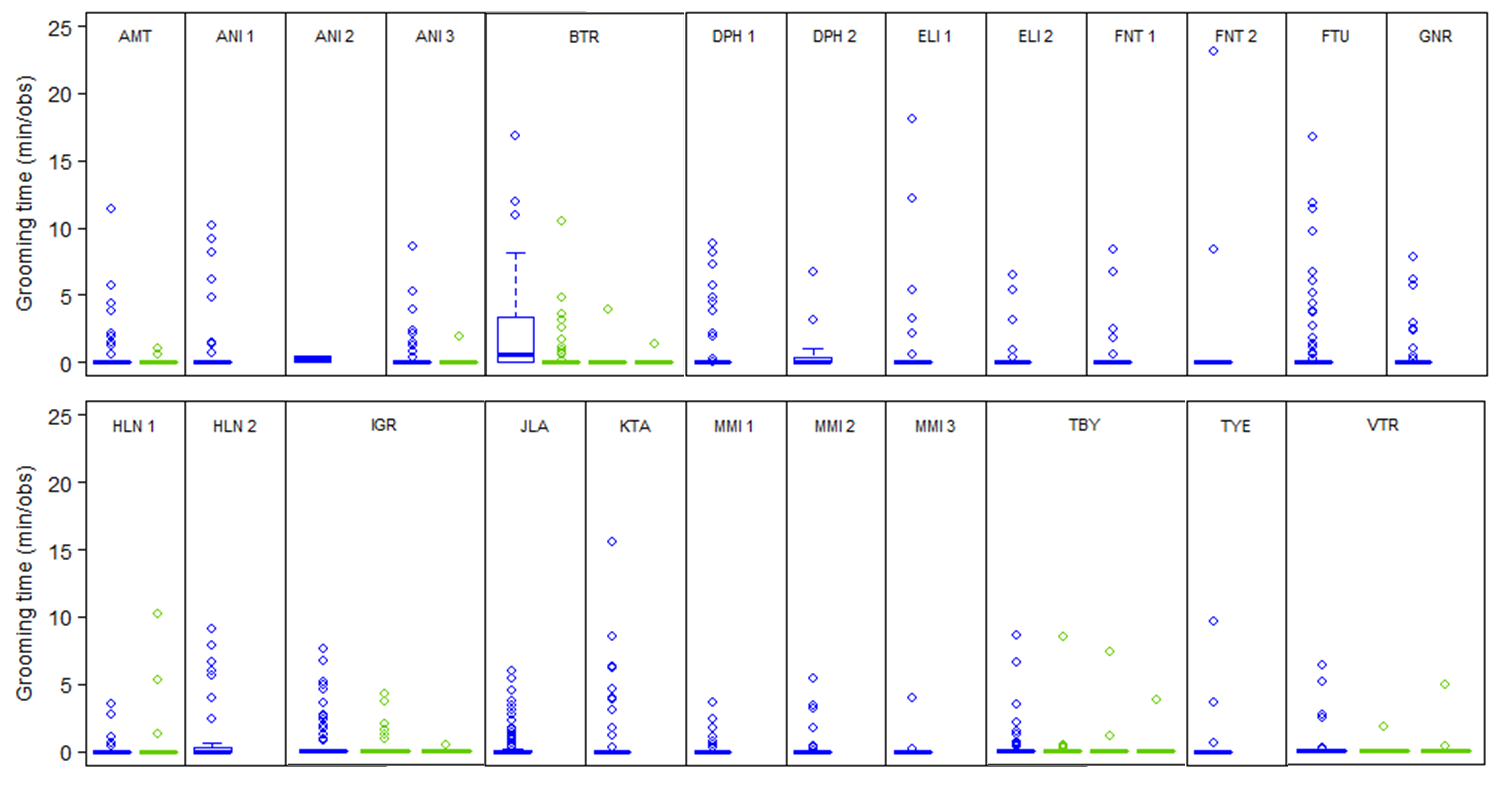


**Fig. S2** The amount of grooming time recorded during focal observations for intersexual dyads, ordered by male status. Each subplot includes that data for one female during the period she was with a specific male; female identities are indicated by the *three letter code* at the top of each subplot and *numbers* differentiate between different OMU periods for transferring females. Primary males are in *blue* and secondary males in *green*. Although spatially available (see Fig. 1 and Fig. S1), not all females were observed to groom with secondary males during focal observation
