# Supplementary material for: Sex and friendship in a multilevel society: behavioural patterns and associations between female and male Guinea baboons
Source: Behav Ecol Sociobiol. 2016 Jan 22;70:323–36. doi: 10.1007/s00265-015-2050-6 (PMC4748025; doi:10.1007/s00265-015-2050-6)
Supplement: Supplementary file 1 — (DOCX 731 kb) [file 265_2015_2050_MOESM1_ESM.docx › RE_ Proofs for your article in Behavioral Ecology and Sociobiology ( 2050 )__AC.pdf]

Springer Customer Support 1

From: Goffe, Adeelia [AGoffe@dpz.eu]
Sent: Tuesday, January 19, 2016 12:52 AM
To: Springer Customer Support 1
Subject: RE: Proofs for your article in Behavioral Ecology and Sociobiology (2050)
Attachments: all_figs.pdf; Fig._1ab_5m_2m_networks.pdf; Fig.2a_Pgroom.pdf; Fig.2b_Pgreet.pdf; Fig.2c_Paggress.pdf; Fig.2d_Pinfhand.pdf; Fig._3_hinde.pdf; Fig._4_Pgreeting_FRS.pdf; Fig._5_temporal_dynamics.pdf; Fig._6_tenure.pdf

Hello,

All figures are also attached here as a pdf.

Best wishes,
Adeelia

-----Original Message-----
From: Goffe, Adeelia
Sent: 16 January 2016 22:07
To: 'CorrAdmin1@spi-global.com'
Subject: RE: Proofs for your article in Behavioral Ecology and Sociobiology (2050)

Dear Mr. Romero,

Thank you for your help with the type setting for my manuscript. I have submitted the changes and I hope that all of my comments are clear.

I would like to point out that I am concerned about the quality of the figures and have therefore added the .png for each figure separately. I believe that the quality of the images were diminished by my putting them into word during the review process.

I hope that it will be possible for you to use these higher quality .png files instead of the original images.

Kind regards,
Adeelia

-----Original Message-----
From: CorrAdmin1@spi-global.com [mailto:CorrAdmin1@spi-global.com]
Sent: 14 January 2016 21:25
To: Goffe, Adeelia
Subject: Proofs for your article in Behavioral Ecology and Sociobiology (2050)

Article Title: Sex and friendship in a multilevel society: behavioural patterns and associations between female and male Guinea baboons
DOI: 10.1007/s00265-015-2050-6
BEAS-D-15-00256.3

Dear Author,

We are pleased to inform you that your paper is nearing publication. Your article proofs are available at:

http://eproofing.springer.com/journals/index.php?token=05uhjh_f0QpIq--CoFd9gzxMHNtF5U3C

The URL is valid only until your paper is published online. It is for proof purposes only and may not be used by third parties.

We hope you are pleased with the publication. You can help us facilitate quick and accurate publication by using our e.Proofing system. The system will show you an HTML version of the article that you can correct online. In addition, you can view/download a PDF version for your reference.

Please submit your corrections within 2 working days and make sure you fill out your response to any AUTHOR QUERIES raised during typesetting. Without your response to these queries, we may not be able to continue with the processing of your article for Online Publication.

Should you encounter difficulties with the proofs, please contact me.

Thank you very much.

Sincerely yours,

Springer Customer Support
SPi Global
LP Information Technology Park,
Jose Romero Sr. St.,
Bagacay, Dumaguete City,
Negros Oriental, 6200 Philippines
e-mail: CorrAdmin1@spi-global.com
Fax: +1-703-5621873
