# Supplementary figures and images for: Sex and friendship in a multilevel society: behavioural patterns and associations between female and male Guinea baboons
Source: Behav Ecol Sociobiol. 2016 Jan 22;70:323–36. doi: 10.1007/s00265-015-2050-6 (PMC4748025; doi:10.1007/s00265-015-2050-6)

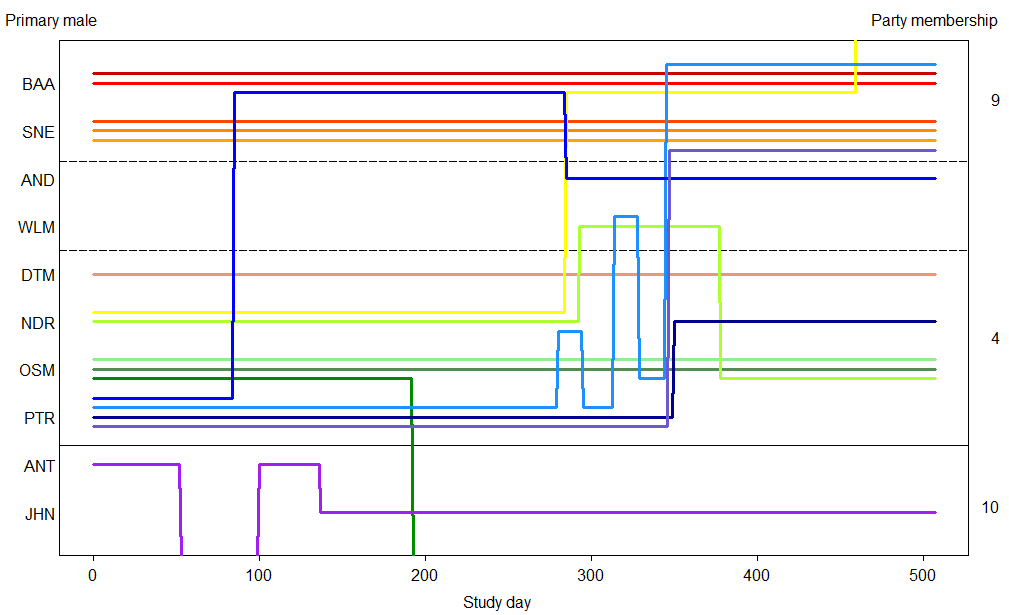

Supplement: Supplementary file 1 — (DOCX 731 kb) [file 265_2015_2050_MOESM1_ESM.docx › upload_1/Fig. 5_temporal_dynamics.png]

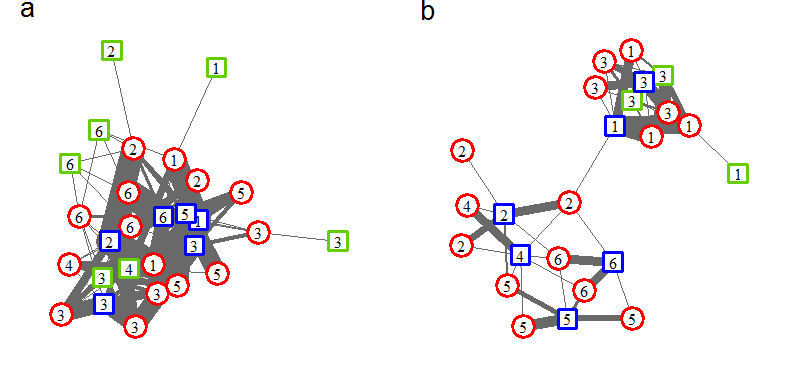

Supplement: Supplementary file 1 — (DOCX 731 kb) [file 265_2015_2050_MOESM1_ESM.docx › upload_1/Fig._1ab_5m_2m_networks.png]

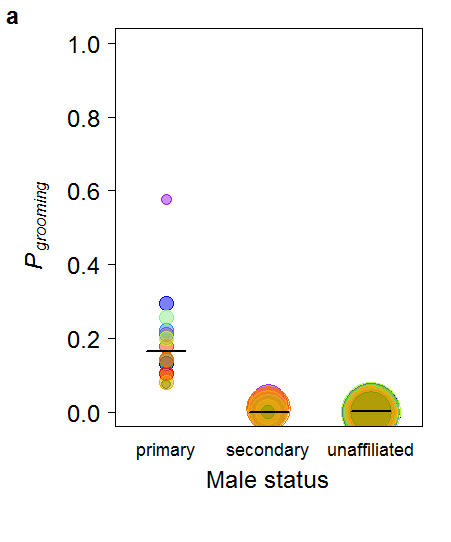

Supplement: Supplementary file 1 — (DOCX 731 kb) [file 265_2015_2050_MOESM1_ESM.docx › upload_1/Fig._2a_Pgroom.png]

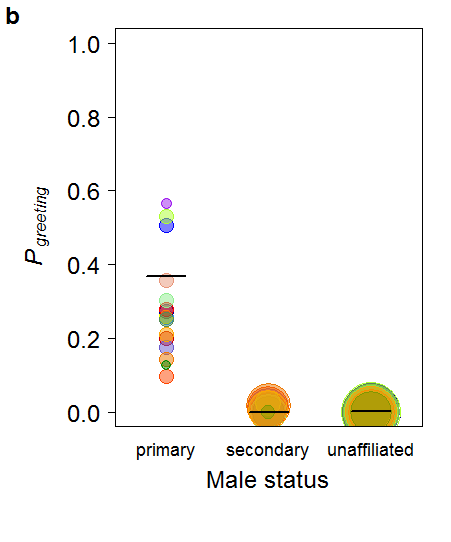

Supplement: Supplementary file 1 — (DOCX 731 kb) [file 265_2015_2050_MOESM1_ESM.docx › upload_1/Fig._2b_Pgreet.png]

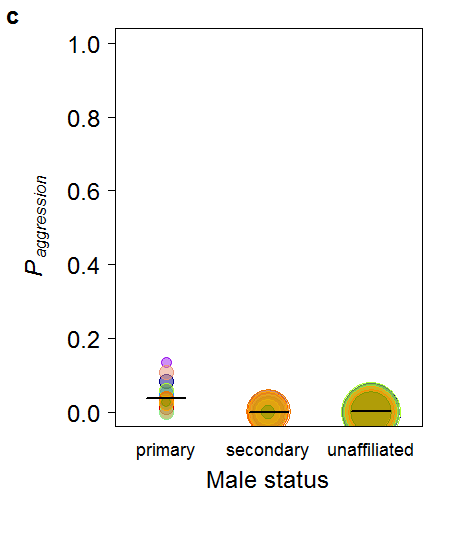

Supplement: Supplementary file 1 — (DOCX 731 kb) [file 265_2015_2050_MOESM1_ESM.docx › upload_1/Fig._2c_Paggress.png]

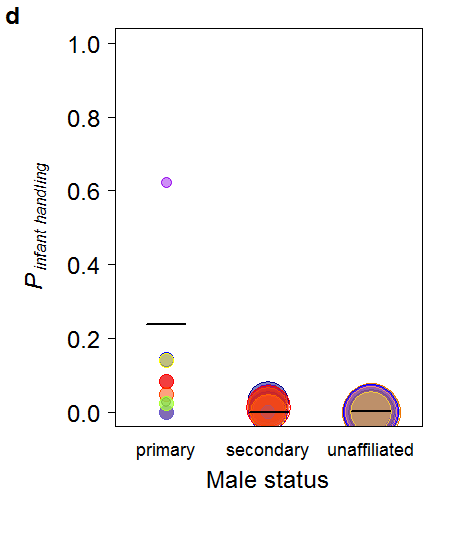

Supplement: Supplementary file 1 — (DOCX 731 kb) [file 265_2015_2050_MOESM1_ESM.docx › upload_1/Fig._2d_Pinfhandle.png]

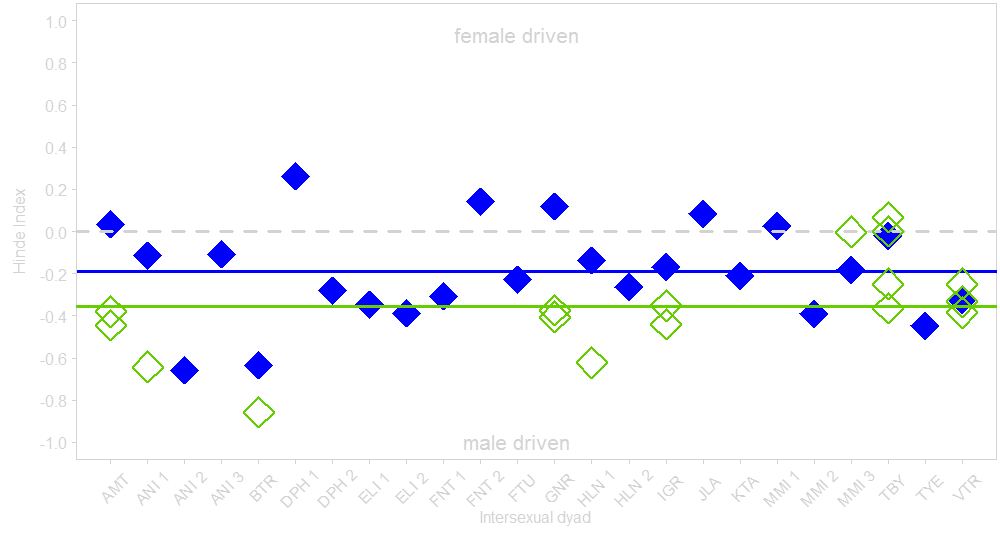

Supplement: Supplementary file 1 — (DOCX 731 kb) [file 265_2015_2050_MOESM1_ESM.docx › upload_1/Fig._3_hinde_ppt.png]

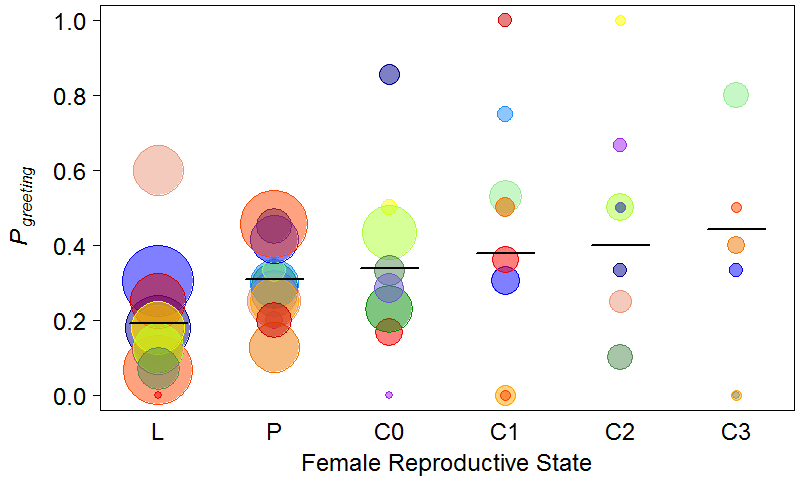

Supplement: Supplementary file 1 — (DOCX 731 kb) [file 265_2015_2050_MOESM1_ESM.docx › upload_1/Fig._4_Pgreeting_FRS.png]

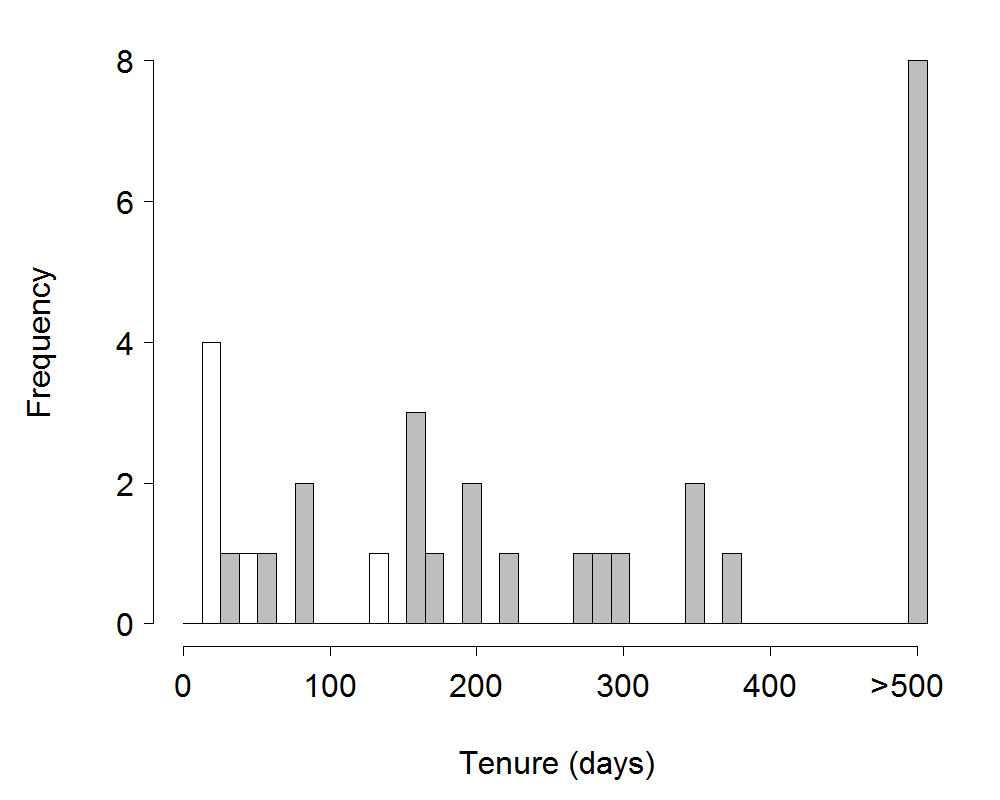

Supplement: Supplementary file 1 — (DOCX 731 kb) [file 265_2015_2050_MOESM1_ESM.docx › upload_1/Fig._6_tenure_mod.png]

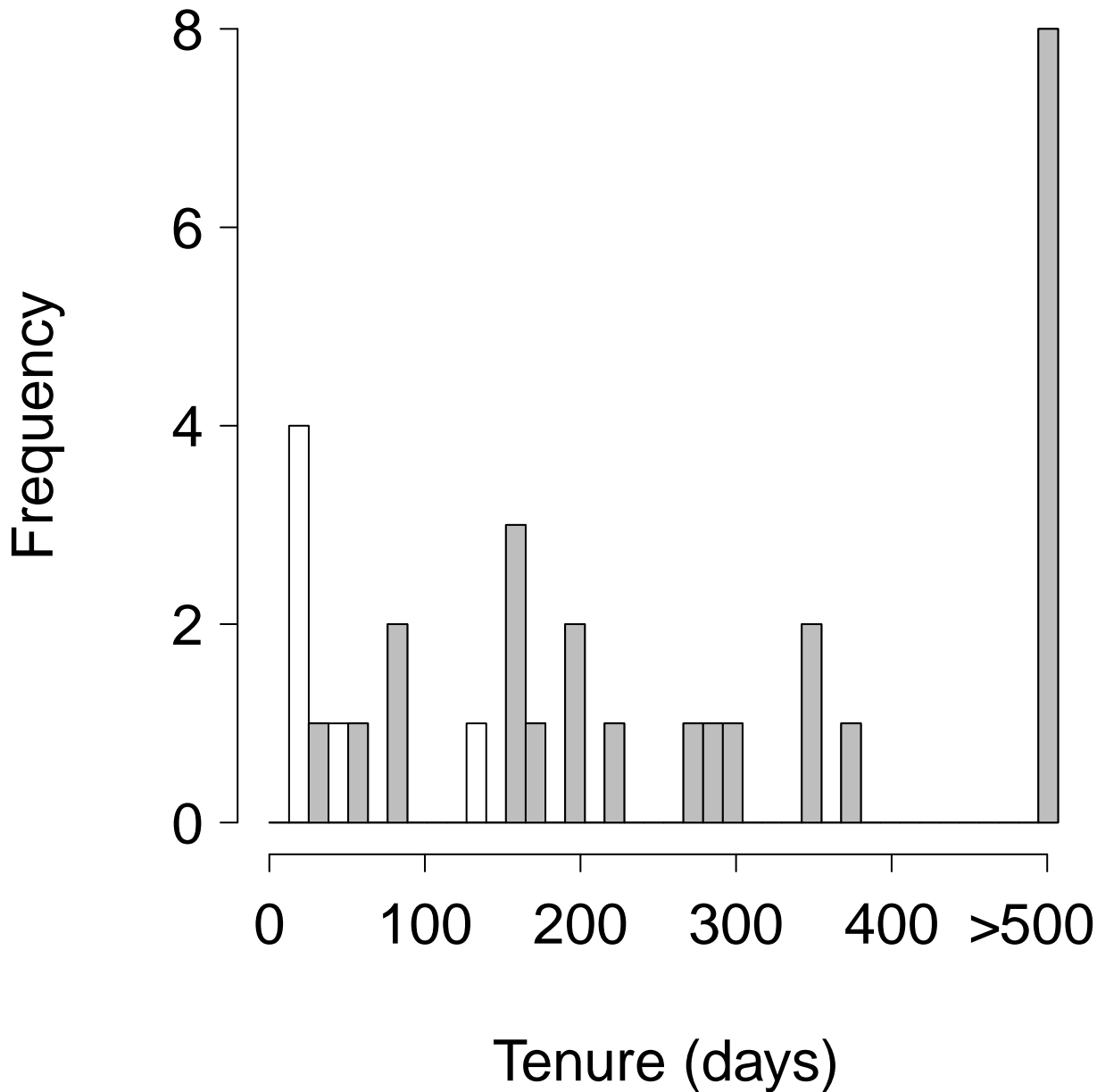

Supplement: Supplementary file 1 — (DOCX 731 kb) [file 265_2015_2050_MOESM1_ESM.docx › Fig._6_tenure_figrep.pdf]

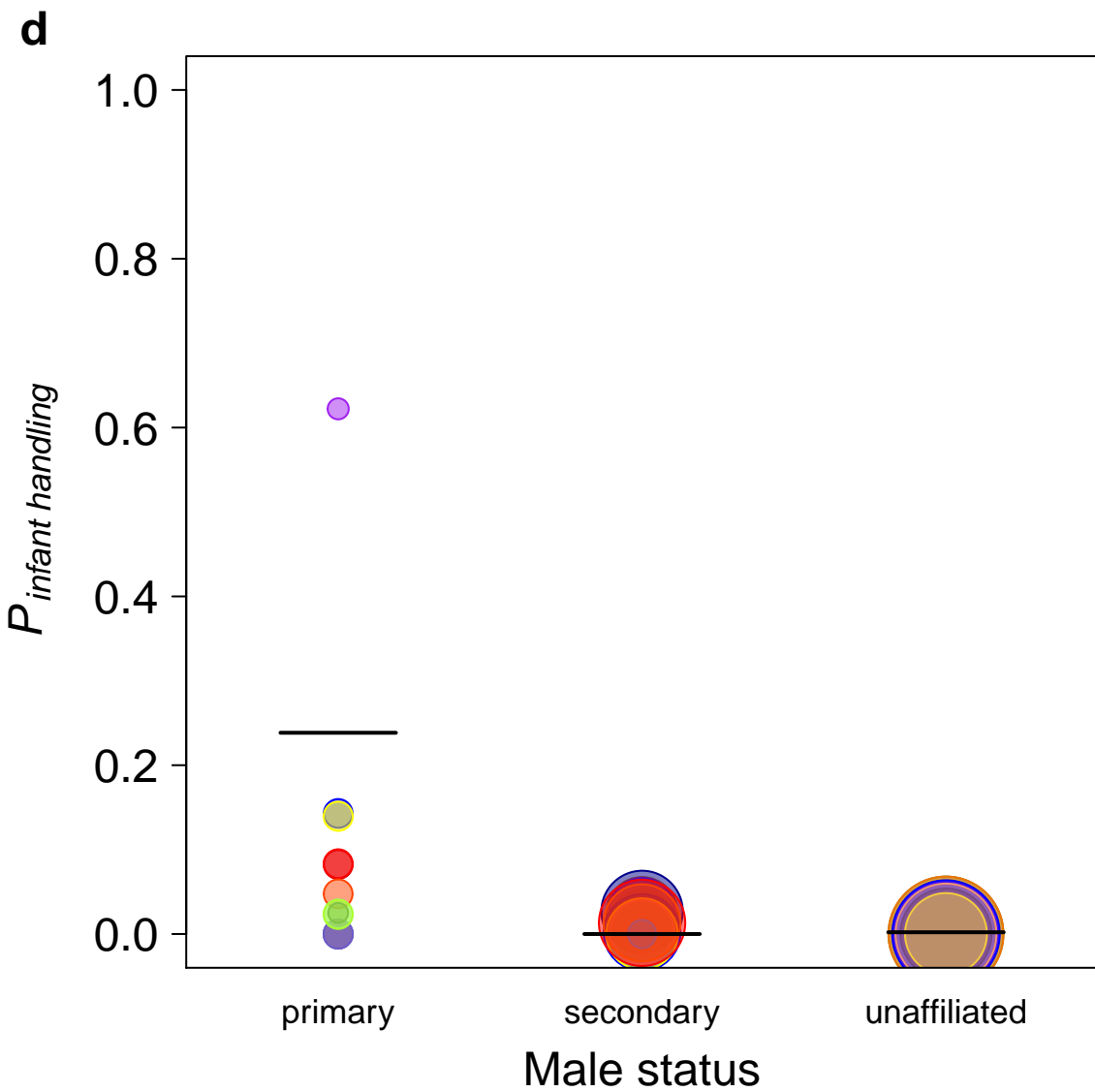

Supplement: Supplementary file 1 — (DOCX 731 kb) [file 265_2015_2050_MOESM1_ESM.docx › Fig.2d_Pinfhand_figrep.pdf]

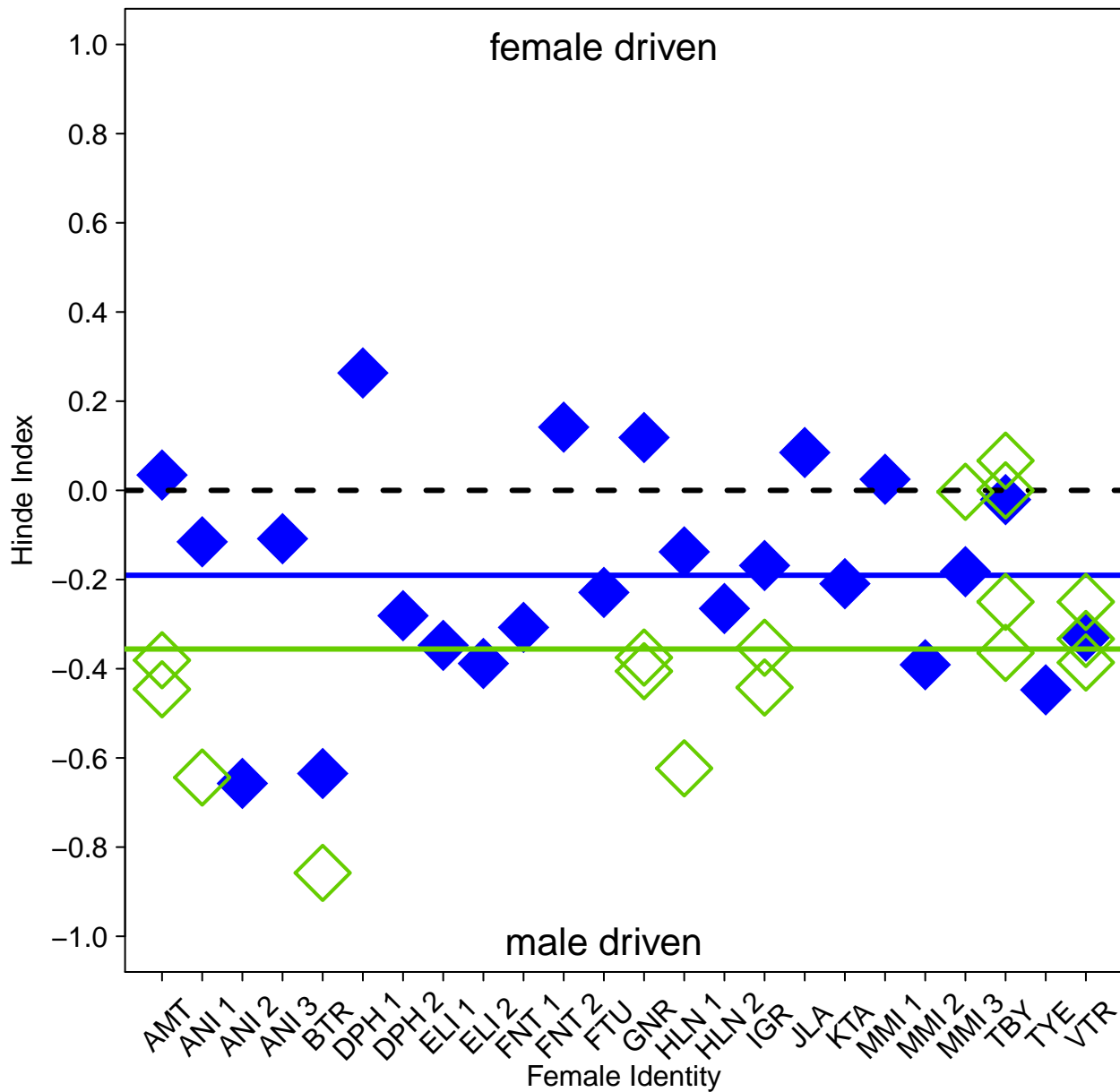

Supplement: Supplementary file 1 — (DOCX 731 kb) [file 265_2015_2050_MOESM1_ESM.docx › Fig._3_hinde_figrep.pdf]

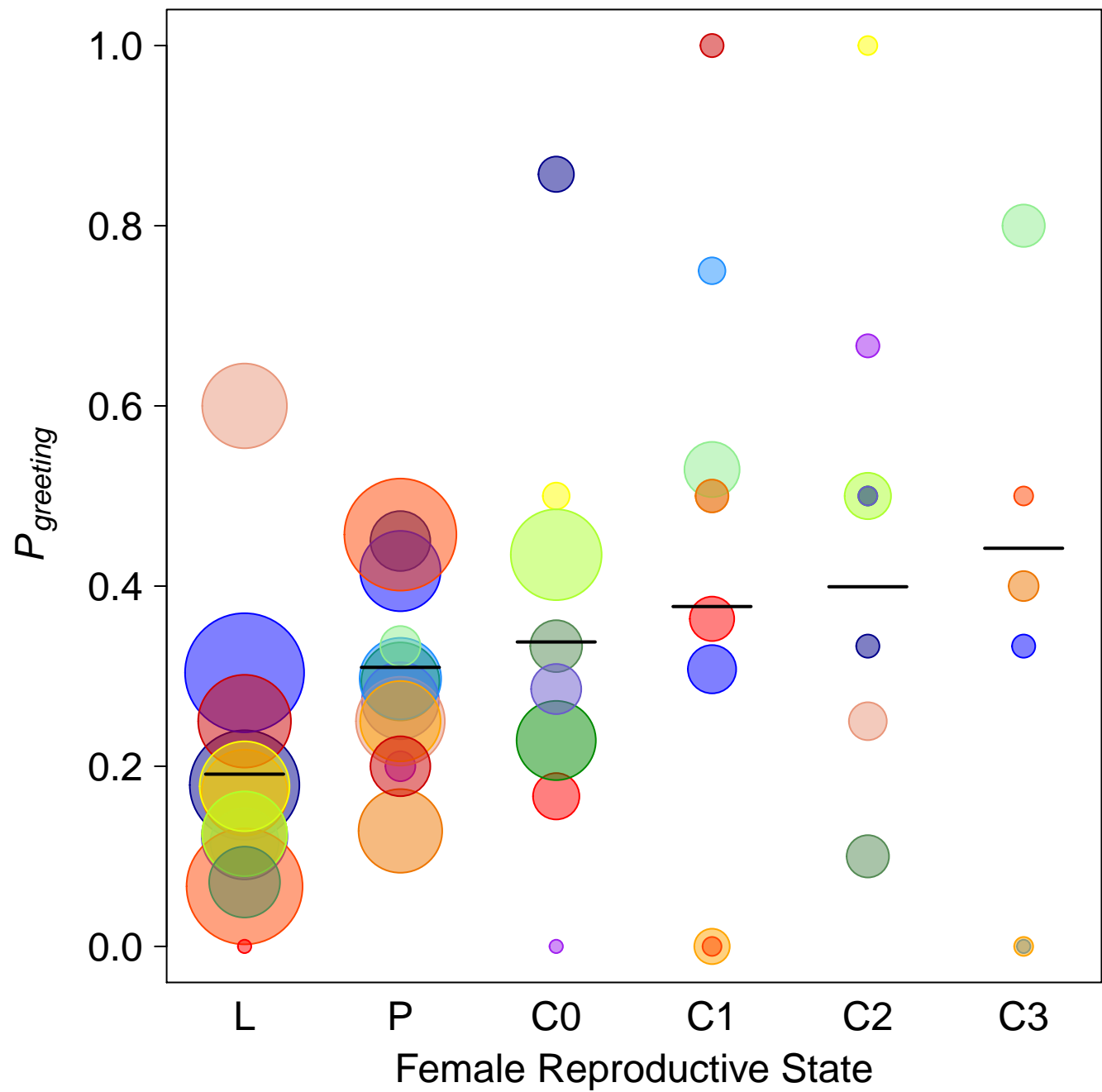

Supplement: Supplementary file 1 — (DOCX 731 kb) [file 265_2015_2050_MOESM1_ESM.docx › Fig._4_Pgreeting_FRS_figrep.pdf]

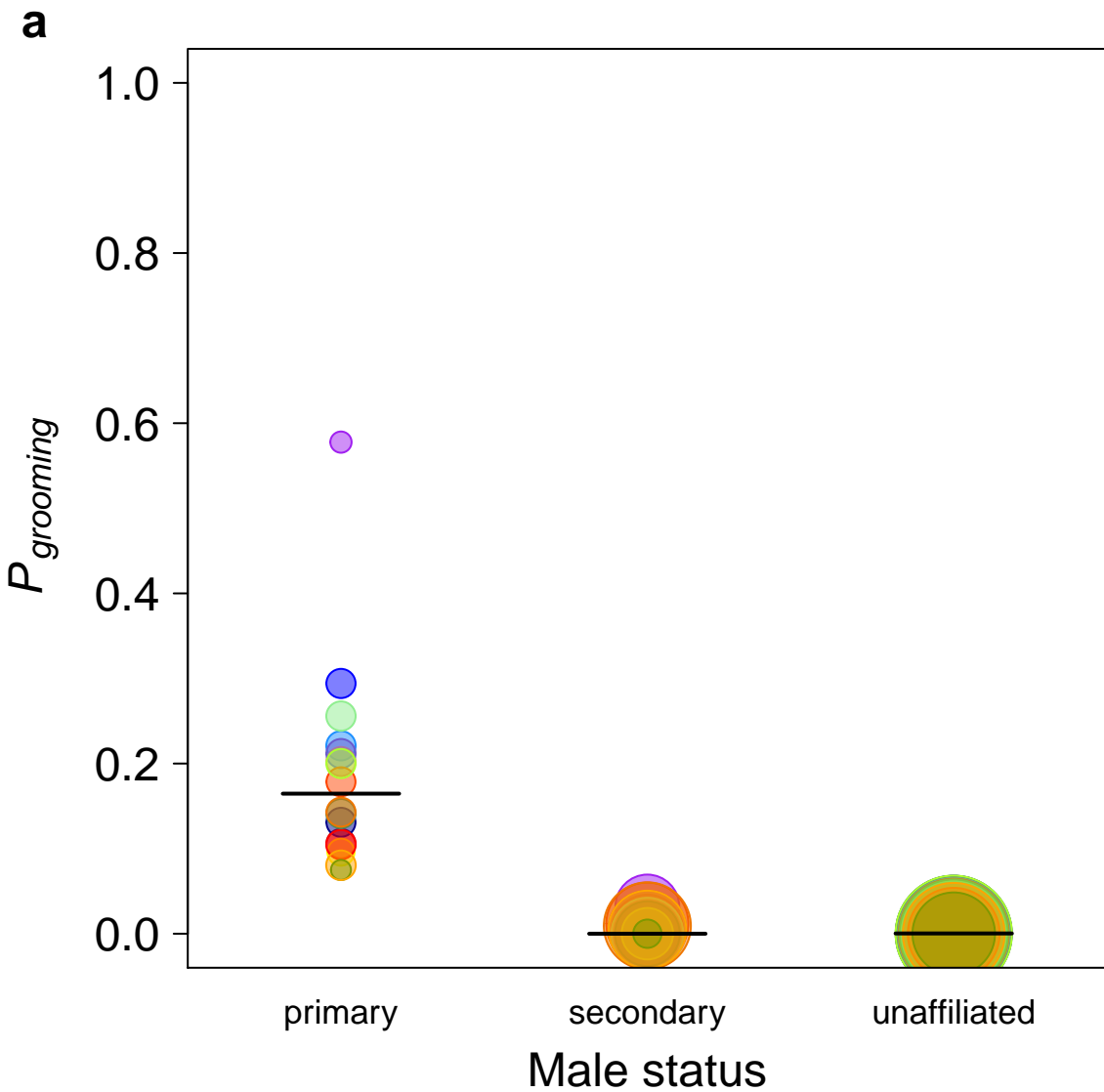

Supplement: Supplementary file 1 — (DOCX 731 kb) [file 265_2015_2050_MOESM1_ESM.docx › Fig.2a_Pgroom_figrep.pdf]

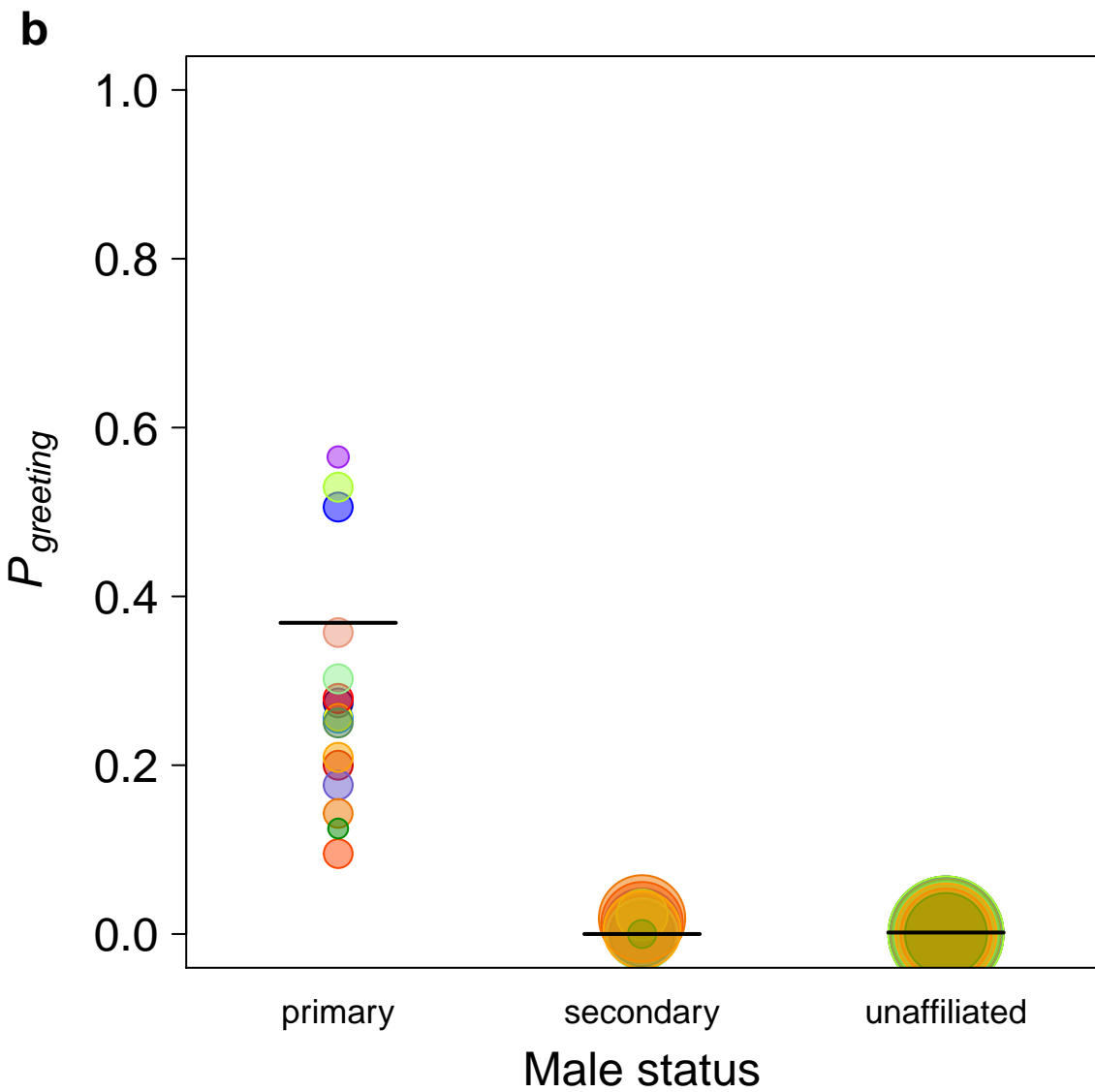

Supplement: Supplementary file 1 — (DOCX 731 kb) [file 265_2015_2050_MOESM1_ESM.docx › Fig.2b_Pgreet_figrep.pdf]

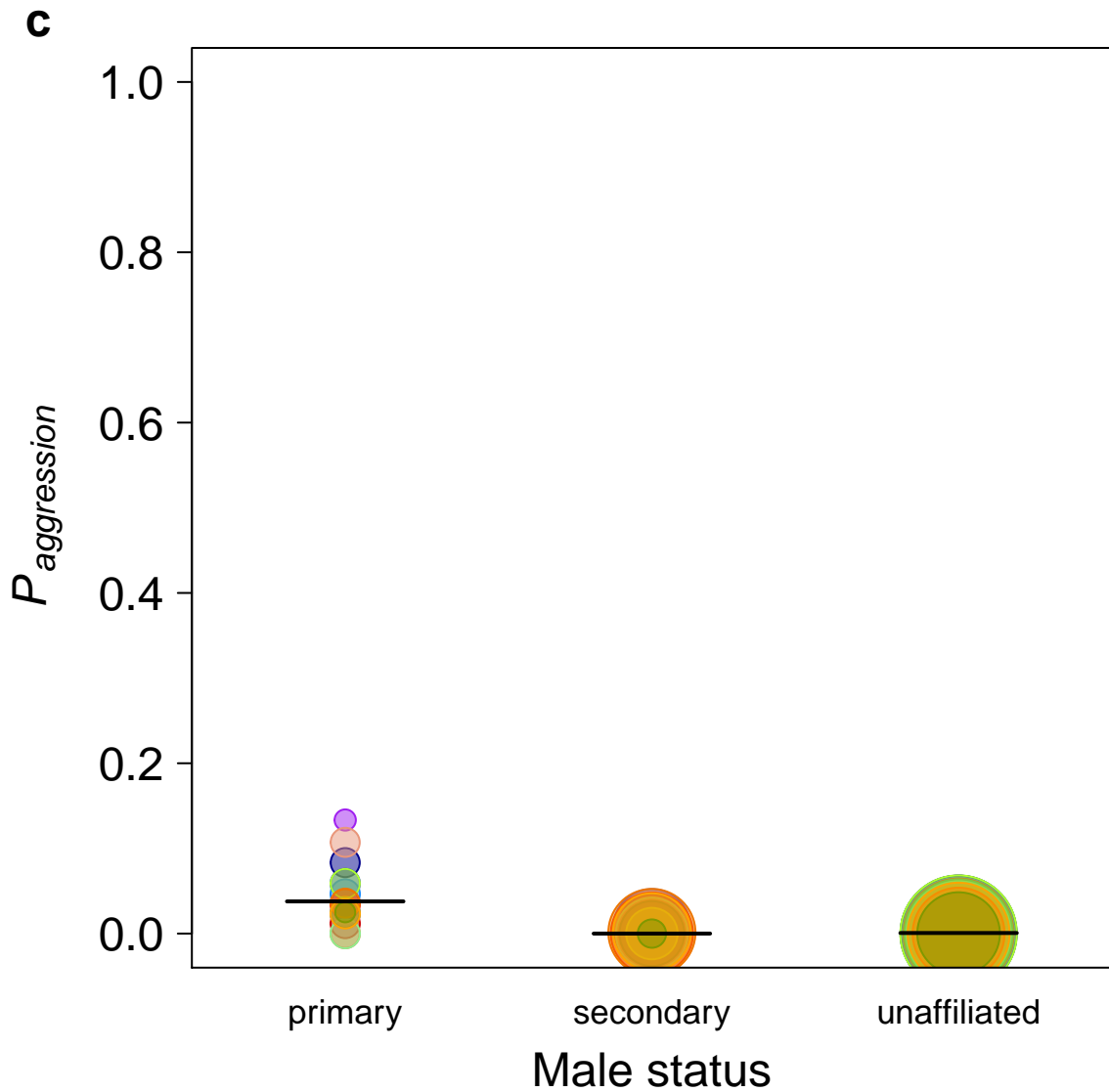

Supplement: Supplementary file 1 — (DOCX 731 kb) [file 265_2015_2050_MOESM1_ESM.docx › Fig.2c_Paggress_figrep.pdf]

a

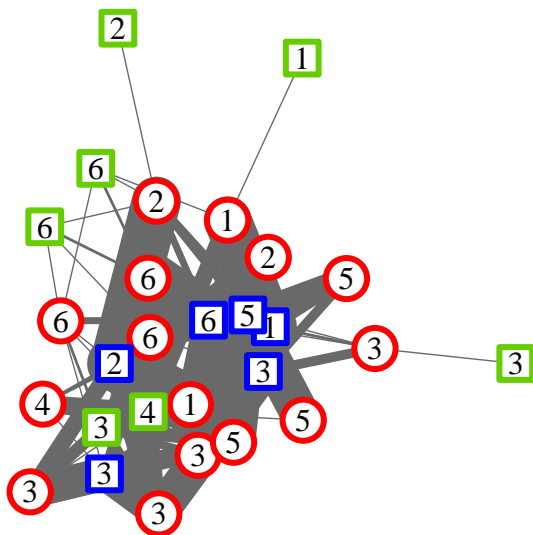

b

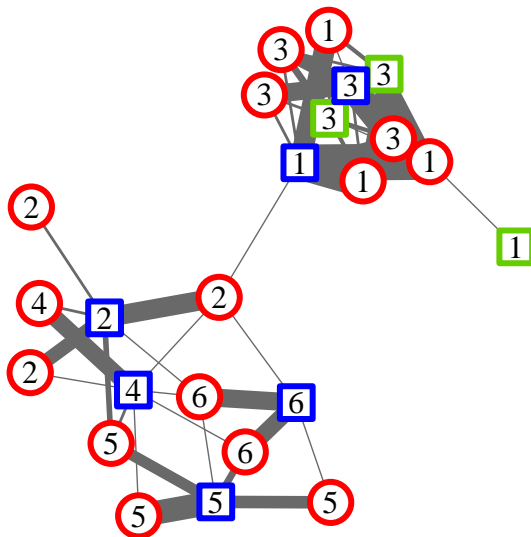

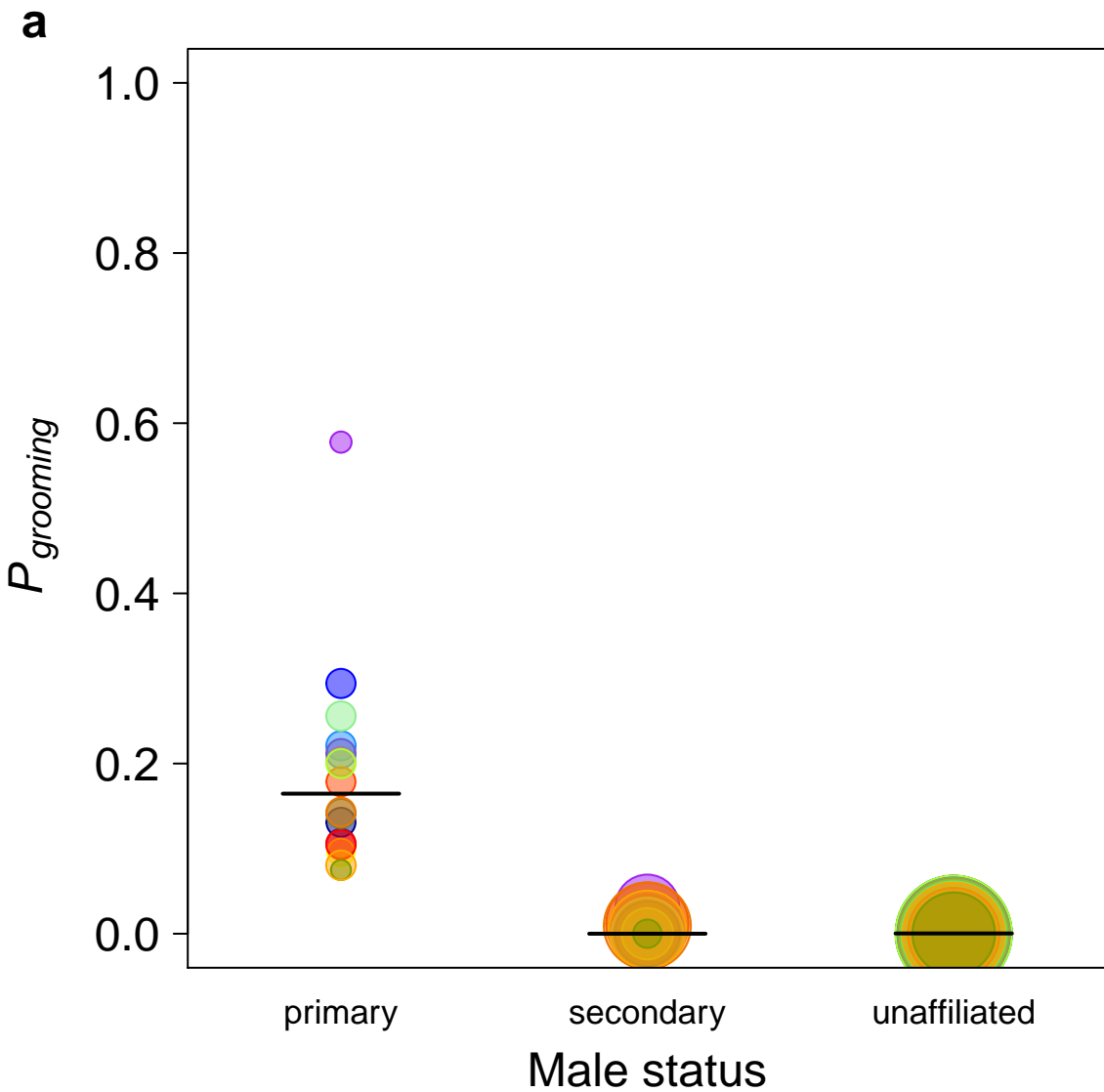

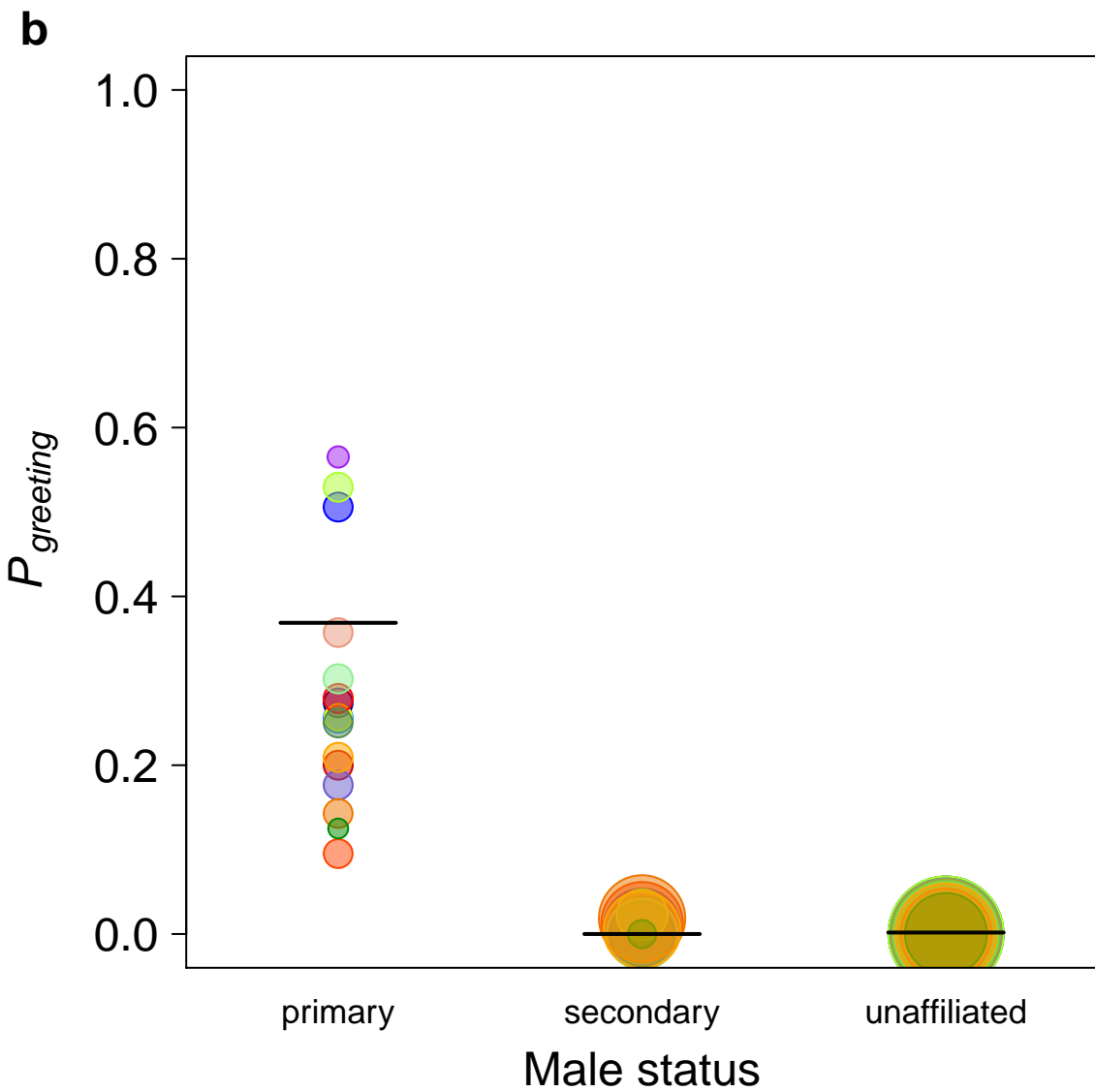

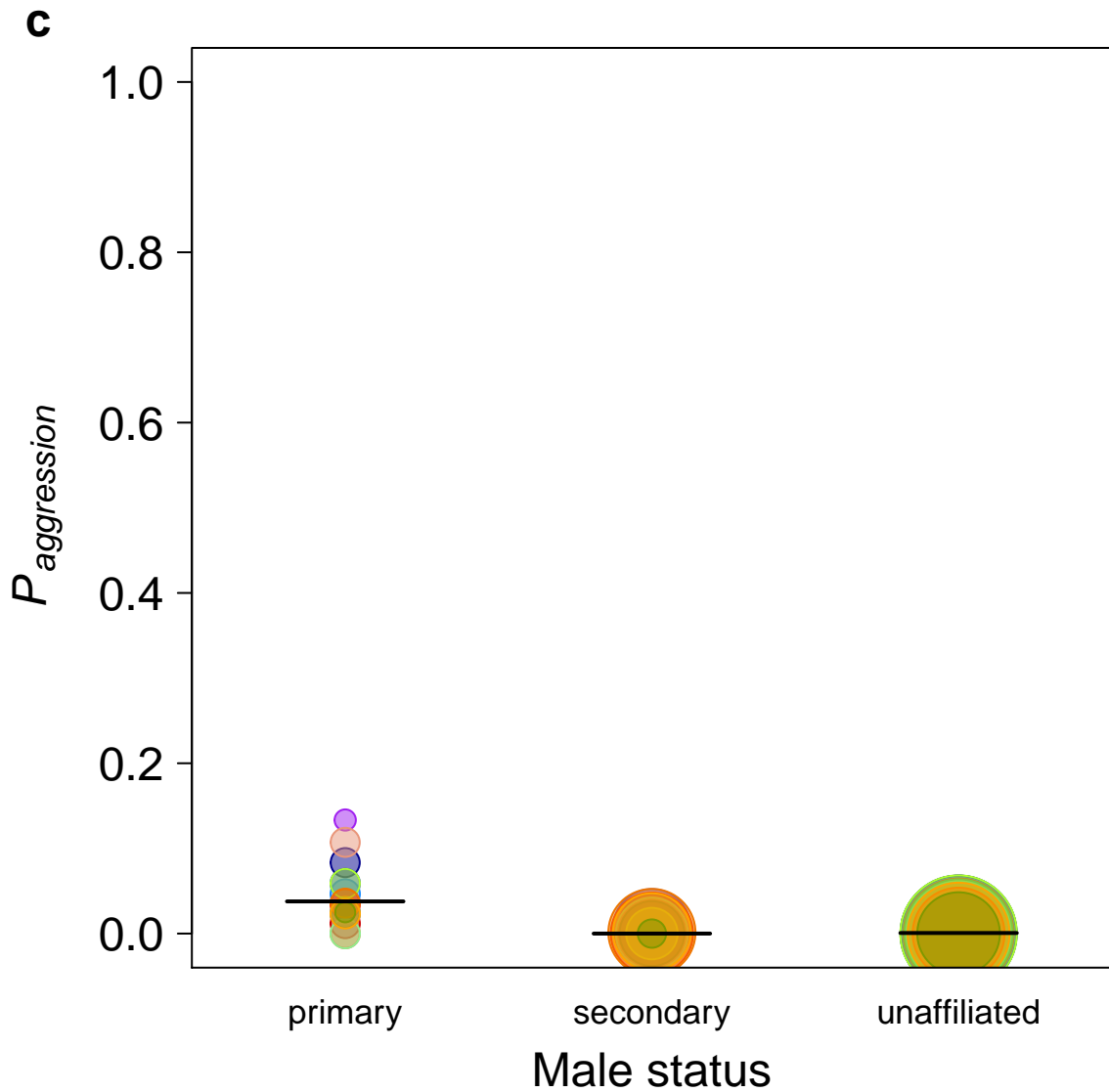

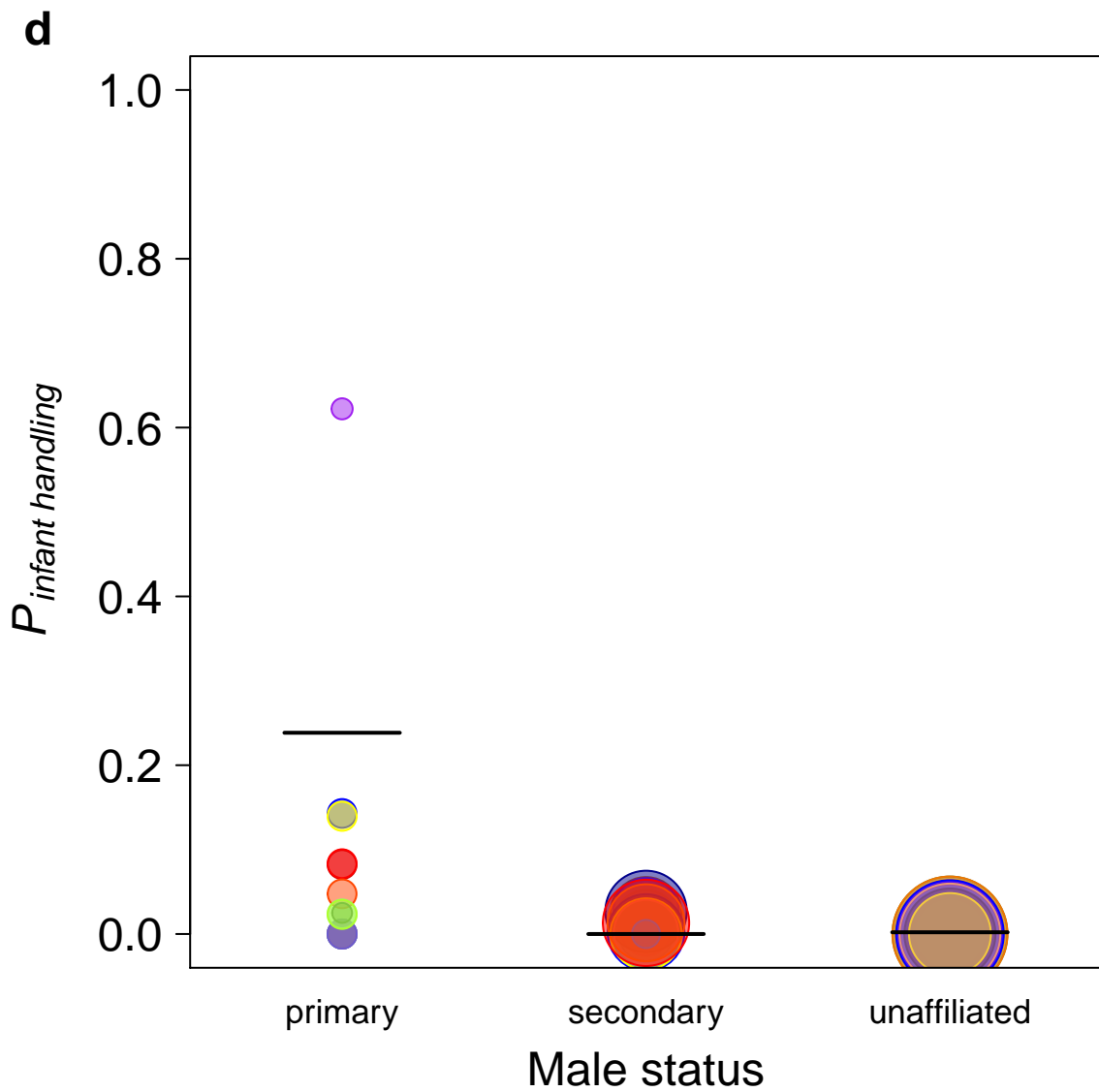

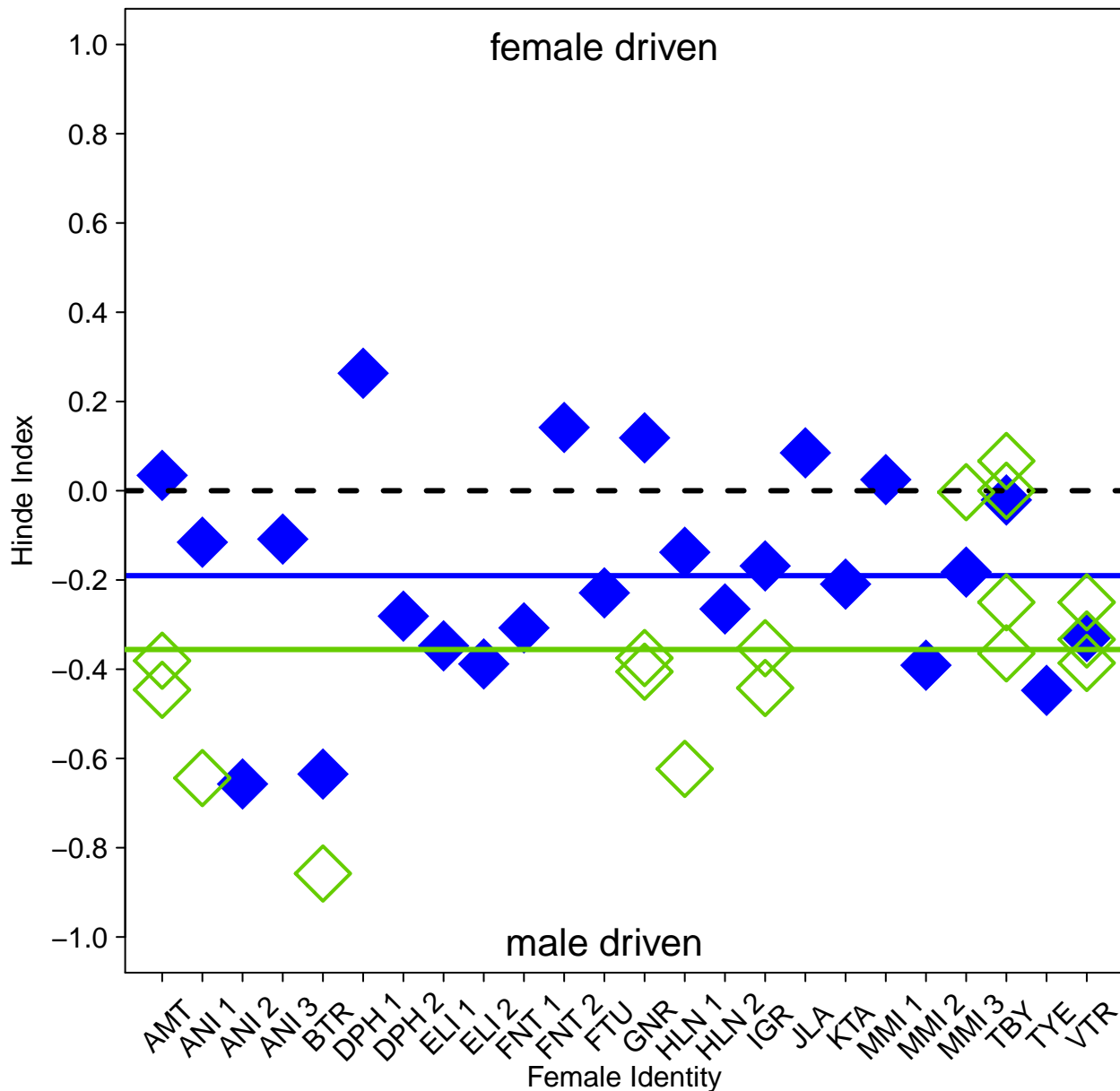

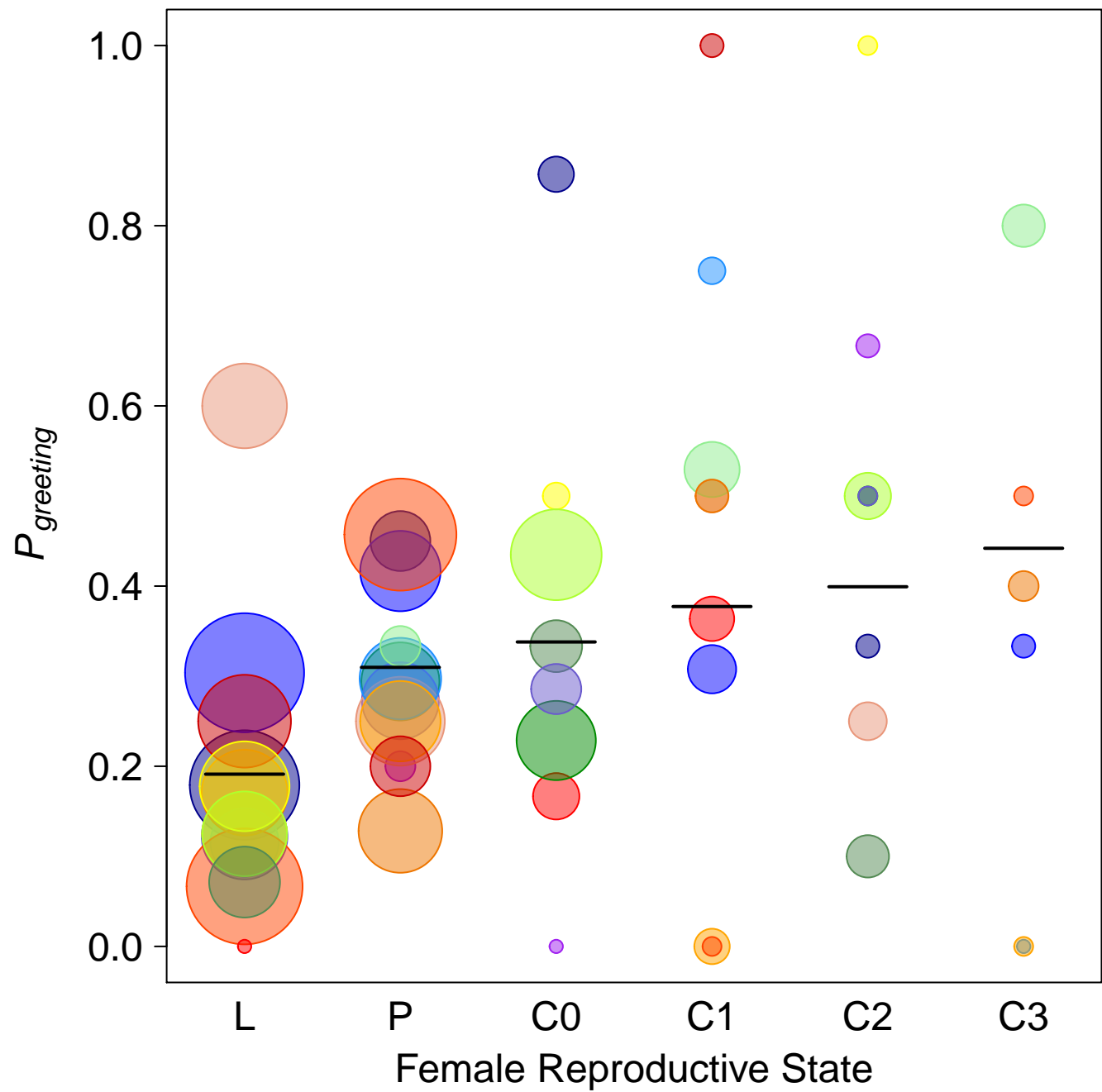

## Party membership

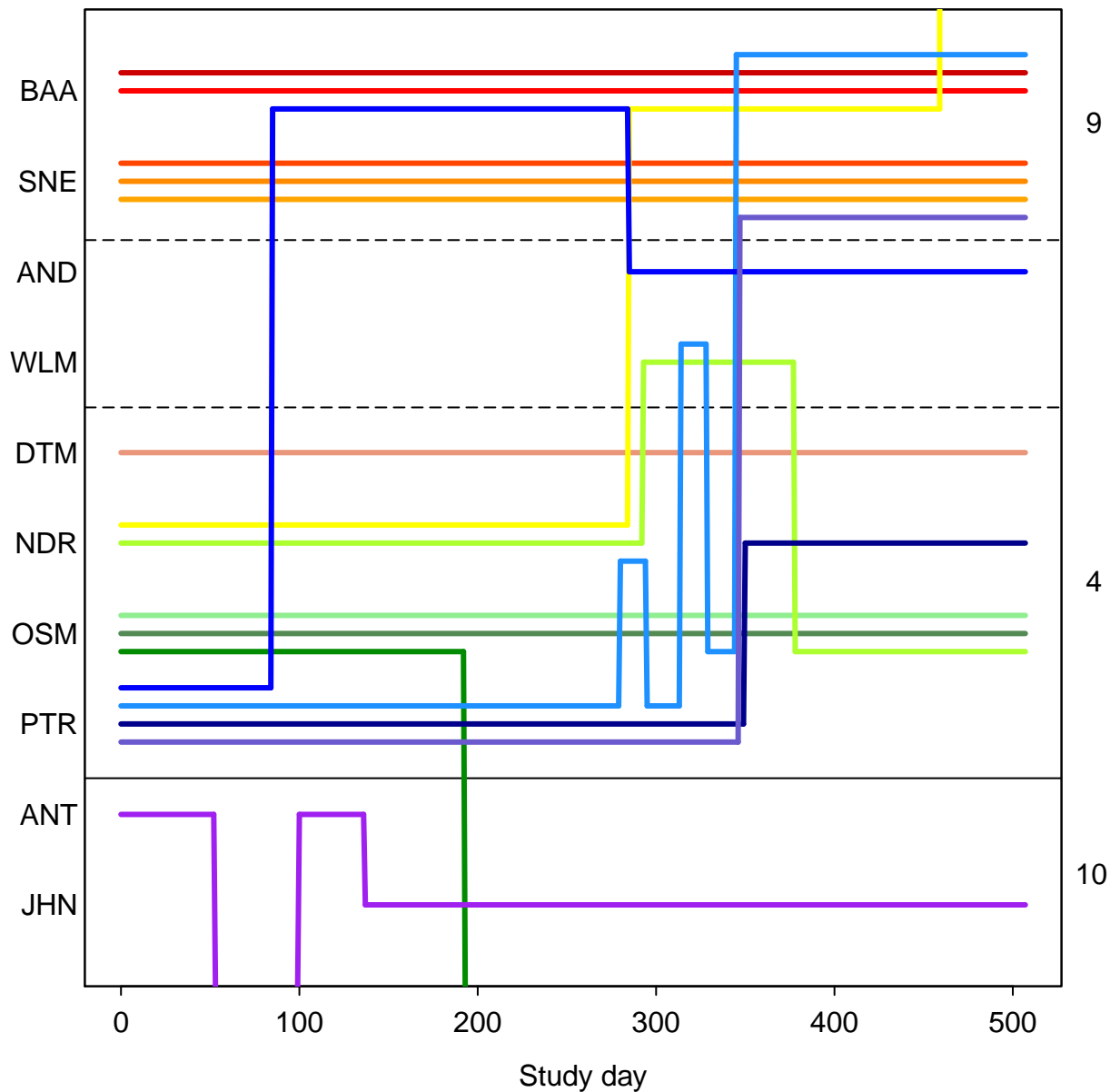

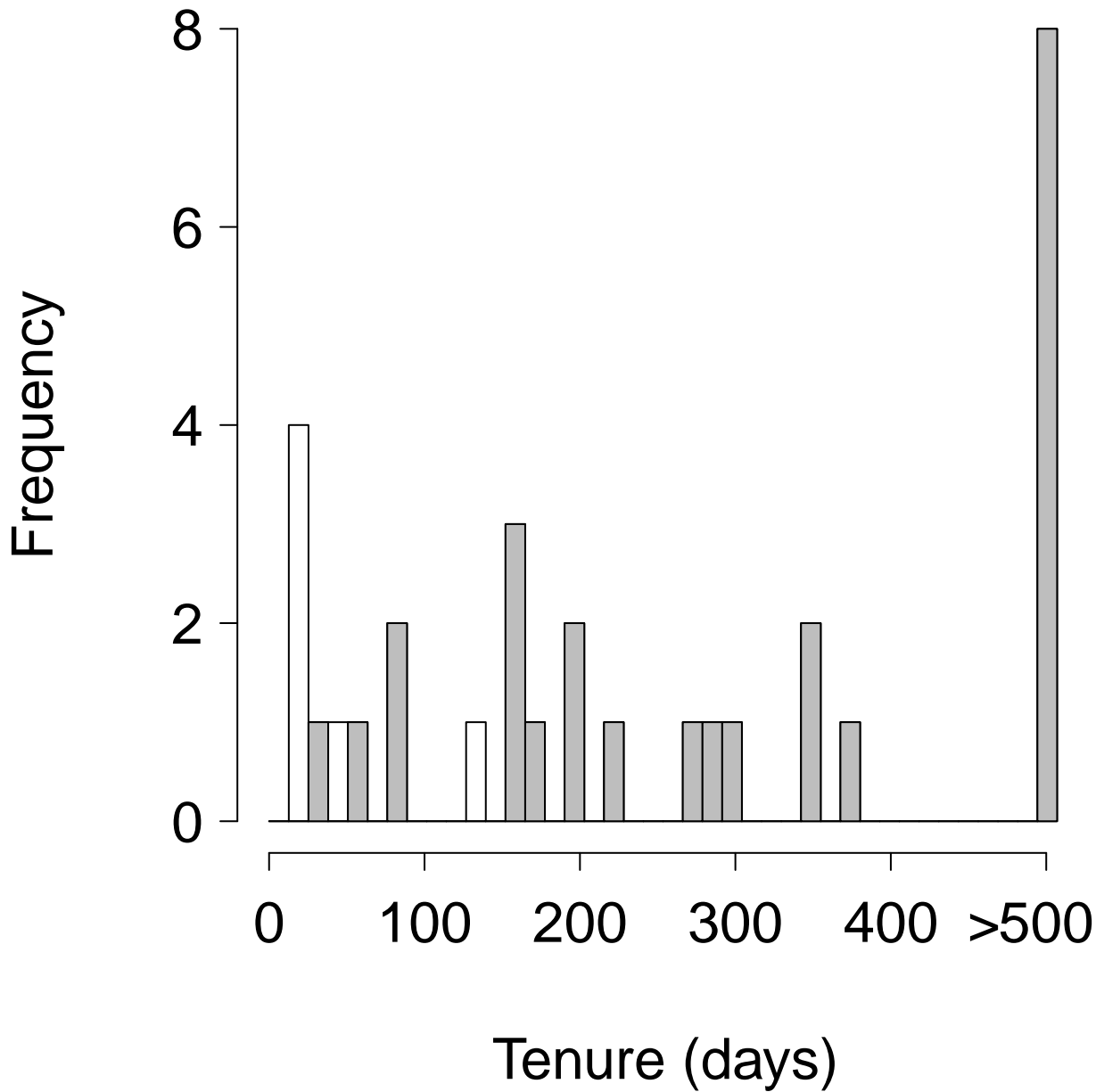

Supplement: Supplementary file 1 — (DOCX 731 kb) [file 265_2015_2050_MOESM1_ESM.docx › all_figs_figrep.pdf]

a

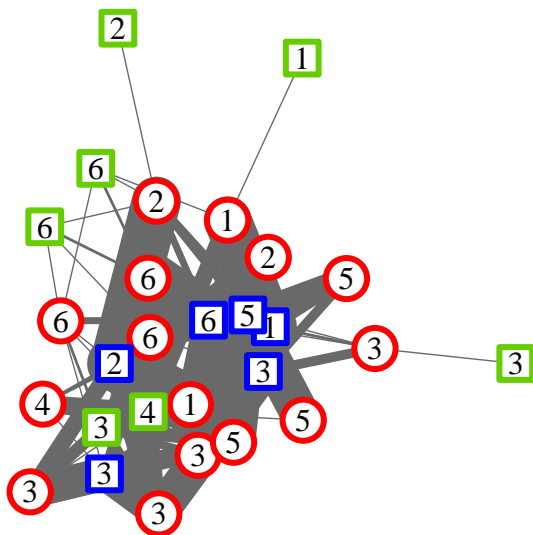

b

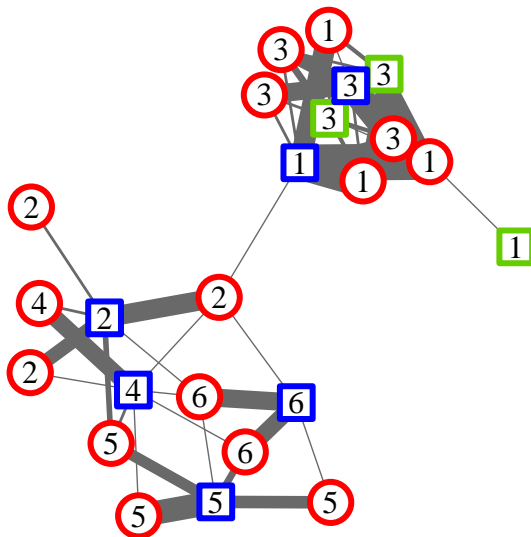

Supplement: Supplementary file 1 — (DOCX 731 kb) [file 265_2015_2050_MOESM1_ESM.docx › Fig._1ab_5m_2m_networks.pdf]
