# Supplementary material for: Sex and friendship in a multilevel society: behavioural patterns and associations between female and male Guinea baboons
Source: Behav Ecol Sociobiol. 2016 Jan 22;70:323–36. doi: 10.1007/s00265-015-2050-6 (PMC4748025; doi:10.1007/s00265-015-2050-6)
Supplement: Supplementary file 1 — (DOCX 731 kb) [file Fig._5_temporal_dynamics_figrep.pdf › Fig._5_temporal_dynamics_figrep.pdf]

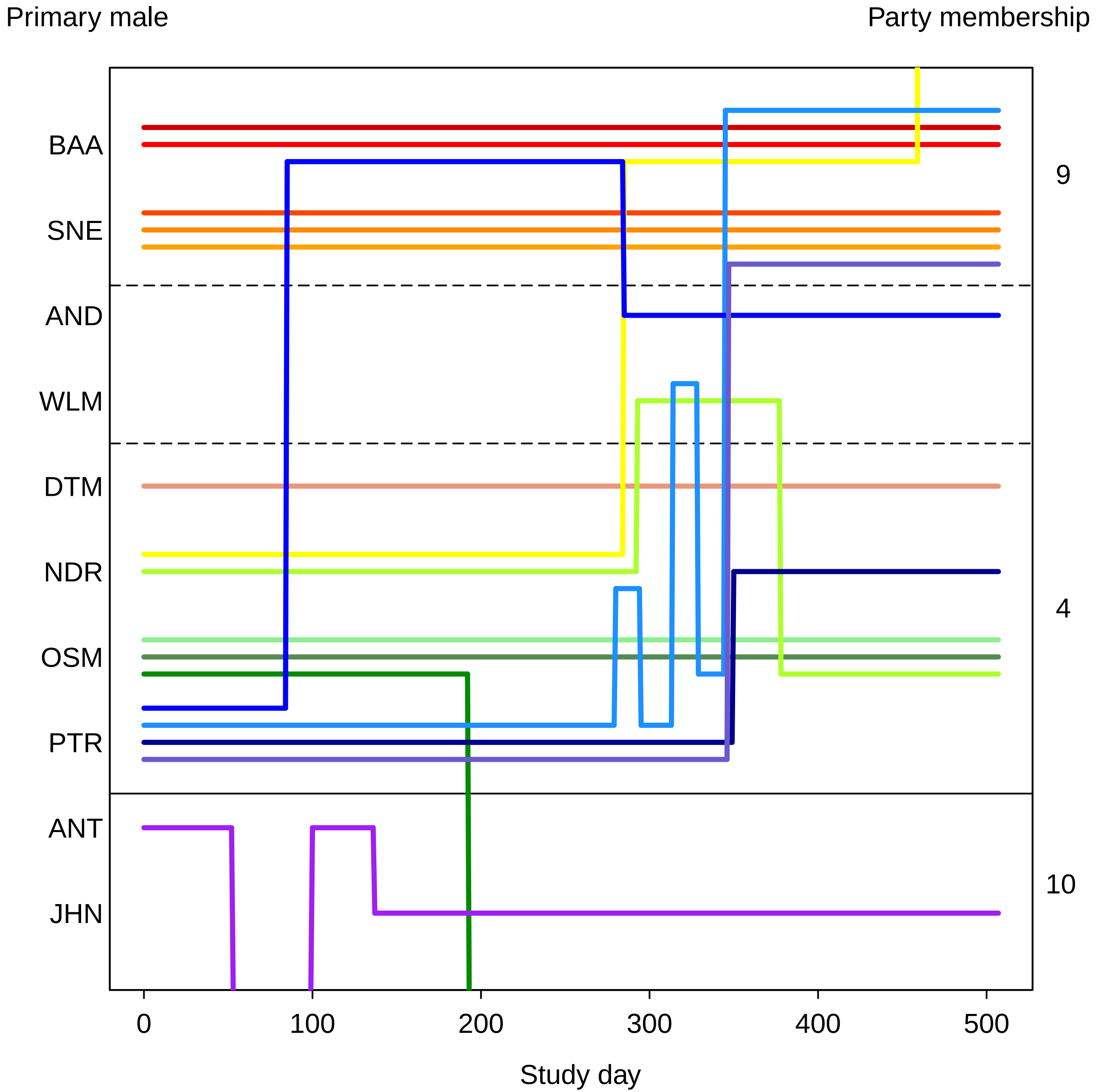
Primary male
Party membership
BAA
SNE
AND
WLM
DTM
NDR
OSM
PTR
ANT
JHN
9
4
10
0
100
200
300
400
500
Study day
